# International survey on the management of portal hypertension

**DOI:** 10.1097/HC9.0000000000001035

**Published:** 2026-08-14

**Authors:** Kiandokht Bashiri, Atoosa Rabiee, Patrick Northup, Shiv Sarin, Don C. Rockey

**Affiliations:** 1 Division of Gastroenterology and Hepatology, Department of Medicine, Baylor College of Medicine, Houston, Texas, USA; 2 Division of Gastroenterology and Hepatology, Department of Medicine, Washington DC VA Medical Center, Washington, District of Columbia, USA; 3 NYU Langone Transplant Institute, New York, New York, USA; 4 Institute of Liver and Biliary Sciences, New Delhi, India; 5 Digestive Disease Research Center, Medical University of South Carolina, South Carolina, USA

**Keywords:** cirrhosis, guidelines, noninvasive assessments, nonselective β-blockers, portal hypertension

## Abstract

**Figure:**
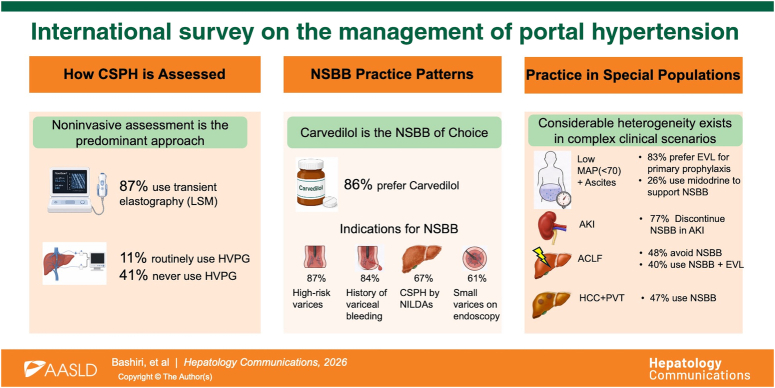

## INTRODUCTION

Portal hypertension in cirrhosis drives severe complications, including variceal hemorrhage, ascites, and HE. Recent guidelines from the American Association for the Study of Liver Diseases (AASLD)^1^ and the Baveno VII consensus^2^ advocate early identification of clinically significant portal hypertension (CSPH) using noninvasive liver disease assessments (NILDAs), such as liver stiffness measurement via transient elastography combined with platelet count thresholds (eg, liver stiffness measurement >25 kPa or 20–25 kPa with platelets <150×10^9^/L). These guidelines position carvedilol as the preferred nonselective β-blocker (NSBB) for primary prophylaxis of variceal bleeding and prevention of decompensation in compensated cirrhosis with CSPH.^1^^–^^3^

Landmark evidence, including the PREDESCI trial, demonstrates that NSBBs in compensated cirrhosis with CSPH reduce the risk of decompensation (eg, ascites, bleeding) or death (HR: 0.51, 95% CI: 0.26–0.97) over 3 years.^4^ Despite these advances, real-world guideline adoption varies, influenced by resource availability, clinician preferences, and uncertainties in special populations like those with refractory ascites, renal dysfunction, or acute illnesses.^5^ Recent expert discussions and surveys reveal persistent heterogeneity, particularly in NSBB management for edge cases where firm guidance is lacking.^5^^,^^6^ Therefore, in order to better understand practitioners’ pragmatic approach to management of portal hypertension, we developed a detailed survey focused on the use of β-blockers, including in difficult clinical scenarios.

## METHODS

A 28-question web-based survey was designed by portal hypertension experts, including members of the AASLD Portal Hypertension Special Interest Group. It covered respondent demographics, HVPG utilization, NILDA preferences, NSBB indications/dosing/monitoring, and management in special populations (eg, ascites, Acute kidney injury (AKI), Acute on chronic liver failure (ACLF), HCC with PVT) (see Supplemental Material, https://links.lww.com/HC9/C469 for the full survey). The survey was disseminated from March to September 2024 to approximately 550 AASLD and APASL members with encouragement of snowball sampling. Participation was voluntary and anonymous. Responses were analyzed descriptively.

## RESULTS

Of 114 respondents, most practiced in the United States, India, China, South Korea, Japan, and Australia (Supplemental Figure S1, https://links.lww.com/HC9/C469). Respondents were predominantly academic (professors 47% [53/114], associate professors 20% [23/114], assistant professors 16% [18/114]) with hepatology (68% [77/114]) or gastroenterology (19% [22/114]) as their primary specialty. Over half (55% [63/114]) saw >30 patients with cirrhosis/portal hypertension monthly (Supplemental Table S1, https://links.lww.com/HC9/C469). Age and gender were not collected.

NILDAs were preferred for CSPH assessment, with liver stiffness measurement via transient elastography being the dominant (87% [85/98]) approach (Table 1). Carvedilol was the favored NSBB (86% [84/98]), with a much smaller proportion of providers preferring propranolol (9% [9/98]) and nadolol (2% [2/98]).

**TABLE 1:** Key survey findings

Category	Practice		n/N (a) (%)
CSPH assessment	Transient elastography (LSM)		85/98 (87)
	Routine clinical HVPG		11/98 (11)
	Never use HVPG		40/98 (41)
NSBB	Preference	Carvedilol	84/98 (86)
	Indications (select all)	High-risk varices	85/98 (87)
		History of variceal bleeding	82/98 (84)
		CSPH by NILDAs	66/98 (67)
		Small varices on endoscopy	60/98 (61)
	Initiation by NILDAs	LSM >25 kPa	63/97 (65)
		LSM 20–25 kPa + platelets <150,000/mm³	56/97 (58)
		LSM 15–20 kPa + platelets <110,000/mm³	50/97 (52)
	Titration and monitoring	Target heart rate 55–60 bpm	67/98 (68)
	Primary prophylaxis	Use NSBB in grade I ascites (imaging)	~51
		Use NSBB in grade II ascites (exam)	~50
Special populations	Low MAP (<70 mm Hg) + ascites	Prefer EVL for primary prophylaxis	73/88 (83)
		Add midodrine to support NSBB	23/88 (26)
	AKI	Discontinue NSBB in AKI (Cr >2 mg/dL), restart postrecovery	68/88 (77)
	ACLF (MELD >20 + ascites + high-risk varices)	Avoid NSBB	43/89 (48)
		Use NSBB + EVL	35/89 (40)
	HCC + PVT	Use NSBB	41/88 (47)

a Note, the “n” reported in response to several questions was lower than the total number of respondents because not all individuals answered every question.

Abbreviations: ACLF, Acute on chronic liver failure; AKI, Acute kidney injury; CSPH, clinically significant portal hypertension; EVL, endoscopic variceal ligation; LSM, liver stiffness measurement; MAP, Mean arterial pressure; NILDA, noninvasive liver disease assessment; NSBB, nonselective β-blocker.

NSBB use for CSPH without high-risk varices was moderate (50%–67%). In patients with ascites and high-risk varices, NSBB utilization remained around 50% in patients with minimal or moderate ascites, consistent with a strong preference for endoscopic variceal ligation in primary prophylaxis (83% [73/88]). Carvedilol was titrated to a target heart rate of 55–60 bpm by most respondents, although heart rate correlates only modestly with HVPG reduction.

In special scenarios we found the following (Table 1): 77% (68/88) discontinued NSBBs in AKI (creatinine >2 mg/dL), restarting postrecovery; 47% (41/88) recommended NSBBs for any-grade varices in HCC with PVT requiring locoregional therapy; 84% (74/88) halted NSBBs post-TIPS if HVPG <10 mm Hg; 48% (43/89) avoided NSBBs in ACLF (MELD >20, ascites, high-risk varices), while 28% (25/89) continued existing ones; in severe AAH without varices, 20% (18/89) used NSBBs; with MELD >20 and likely portal hypertension, 40% (36/89) used NSBB+steroids.

## DISCUSSION

This survey demonstrates robust conformity with AASLD/Baveno VII guidelines,^1^^,^^2^ including widespread NILDA adoption and carvedilol preference, which aligns with evidence from the PREDESCI trial and carvedilol meta-analyses, but reveals practice variability in complex scenarios.^3^^,^^4^

Limited HVPG use (11% [11/98] regular) aligns with its constraints in nontertiary settings, favoring NILDAs like transient elastography (87% [85/98]), consistent with guideline-endorsed thresholds for CSPH (sensitivities >85% in meta-analyses).^1^^,^^2^^,^^7^

NSBBs were used cautiously in decompensated patients with ascites, with endoscopic variceal ligation often preferred when the mean arterial pressure is low. Discontinuation of NSBBs in patients with AKI was common (77%). Practices in ACLF, severe alcohol-associated hepatitis, and HCC with PVT were individualized, possibly reflecting limited high-quality evidence in these populations.

We recognize limitations of this study, including the low response rate (<21% of invited participants), snowball sampling (likely favoring academic experts from the United States and Asia), the fact that not all respondents answered all questions, and the inability to perform US versus Asia subgroup analyses due to dataset constraints. Self-reporting may also have led to an overestimate of guideline adherence. Despite these limitations, the results provide a valuable benchmark for guideline uptake and highlight priority areas for future research, including optimized management in decompensated cirrhosis and special populations.

In summary, the findings highlight several important gaps between guideline recommendations and real-world practice and identify areas for future investigation and implementation efforts.

## Supplementary Material
