# Supplementary material for: International survey on the management of portal hypertension
Source: Hepatol Commun. 2026 Aug 14;10(9):e1035. doi: 10.1097/HC9.0000000000001035 (PMC13480869; doi:10.1097/HC9.0000000000001035)
Supplement: Supplementary file 1 [file hc9-10-e1035-s001.pdf]

Supplemental Material - International Survey on the Management of Portal Hypertension: A Research Letter (Bashiri, et al.)

I. Figure and Table

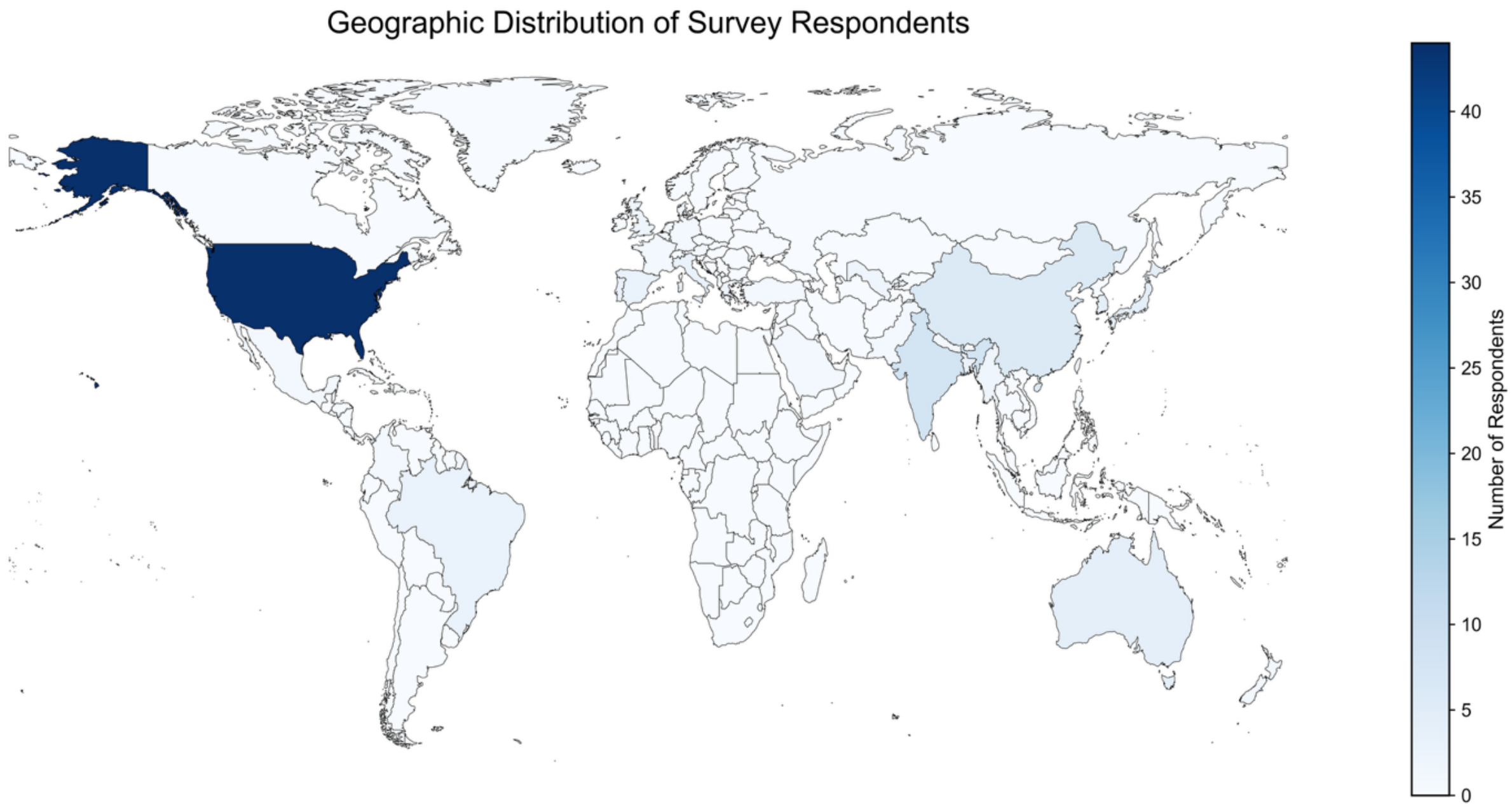

Supplemental Figure 1. Choropleth world map showing the country-level distribution of clinicians who completed the international survey on the use of non-selective beta-blockers in portal hypertension (N = 114). Countries are shaded according to the number of respondents, with darker colors indicating higher participation. The United States contributed the largest proportion of respondents, followed by India, China, South Korea, Japan, and Australia.

Supplemental Table 1- Respondent characteristics (n = 114).

Professional Title	
Trainee	4%
Assistant Professor	16%
Associate Professor	20%
Professor	46%
Other	13%
Specialty	
Internal Medicine	3%
Gastroenterology	19%
Hepatology	68%
Surgeon	4%
Other	6%

II. SurveyMonkey

Q1 Country of Practice

Answered: 114 Skipped: 0

Q2 Professional Title

Answered: 114 Skipped: 0

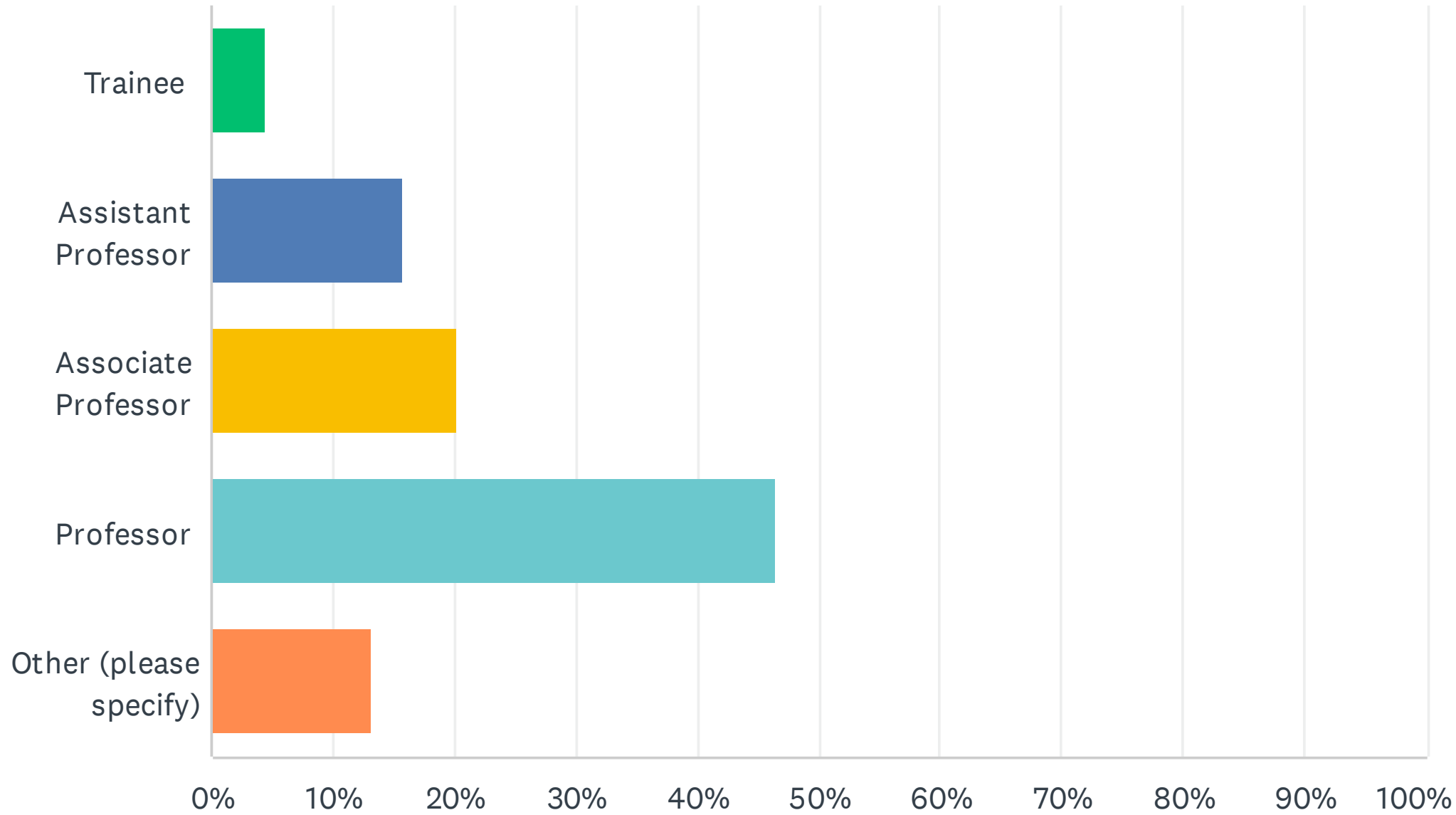

ANSWER CHOICES	RESPONSES	
Trainee	4.39%	5
Assistant Professor	15.79%	18
Associate Professor	20.18%	23
Professor	46.49%	53
Other (please specify)	13.16%	15
TOTAL		114

Q3 Specialty

Answered: 114 Skipped: 0

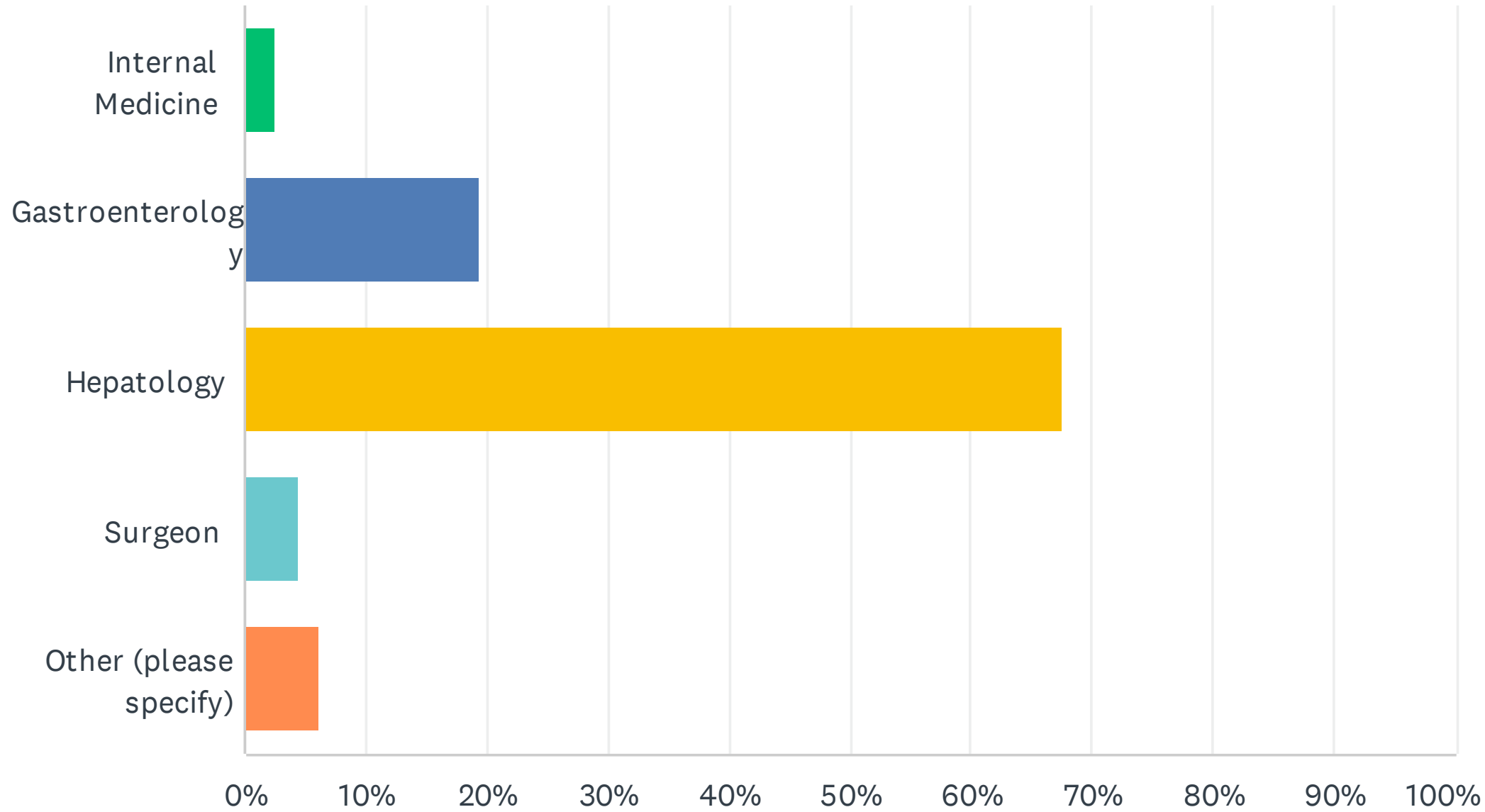

ANSWER CHOICES	RESPONSES	
Internal Medicine	2.63%	3
Gastroenterology	19.30%	22
Hepatology	67.54%	77
Surgeon	4.39%	5
Other (please specify)	6.14%	7
TOTAL		114

Q4 Approximately how many patients with cirrhosis and PHT do you see on average every month?

Answered: 114 Skipped: 0

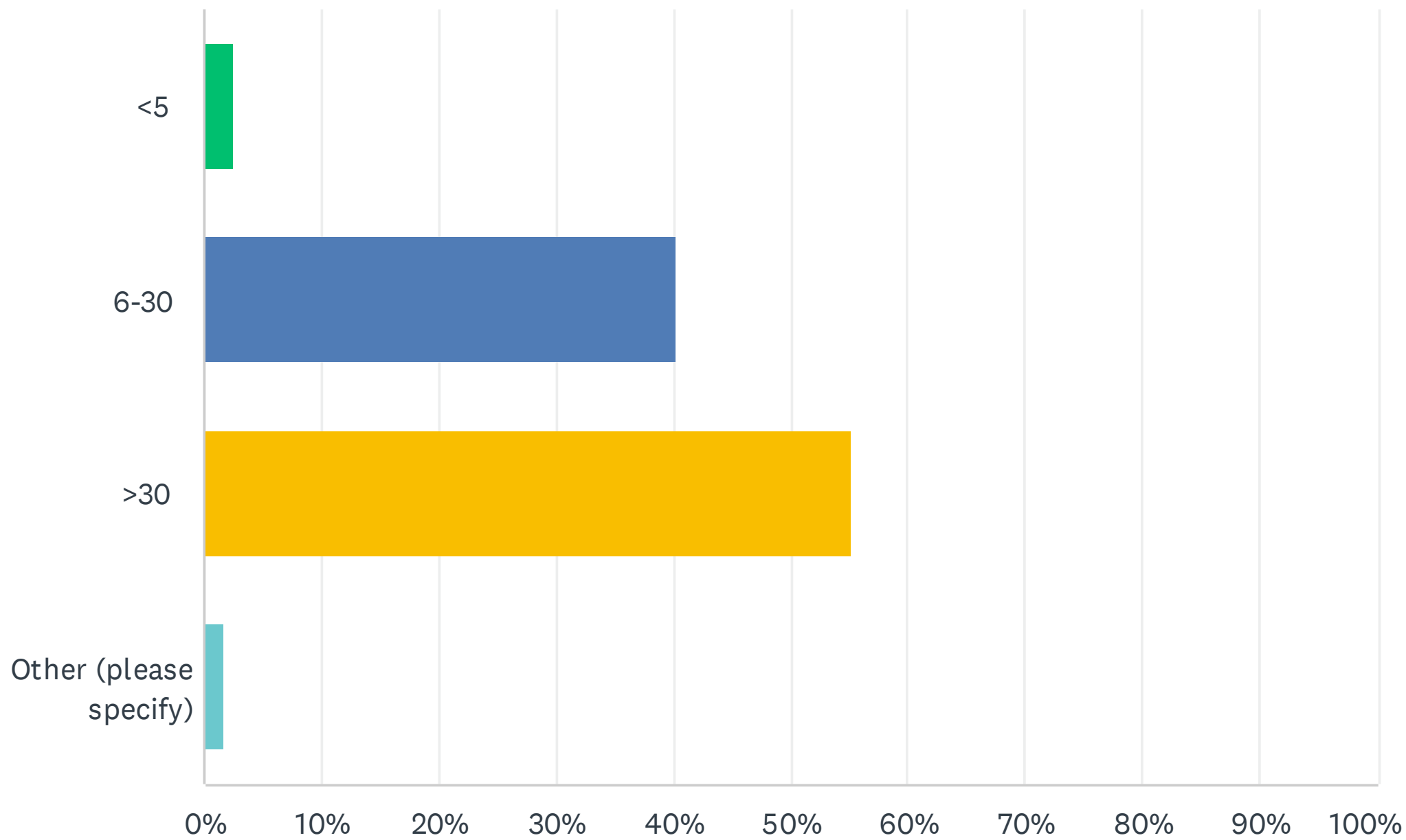

ANSWER CHOICES	RESPONSES	
<5	2.63%	3
6-30	40.35%	46
>30	55.26%	63
Other (please specify)	1.75%	2
TOTAL		114

Q5 Do you use HVPG measurement regularly in clinical practice to assess the severity of PHT

Answered: 98 Skipped: 16

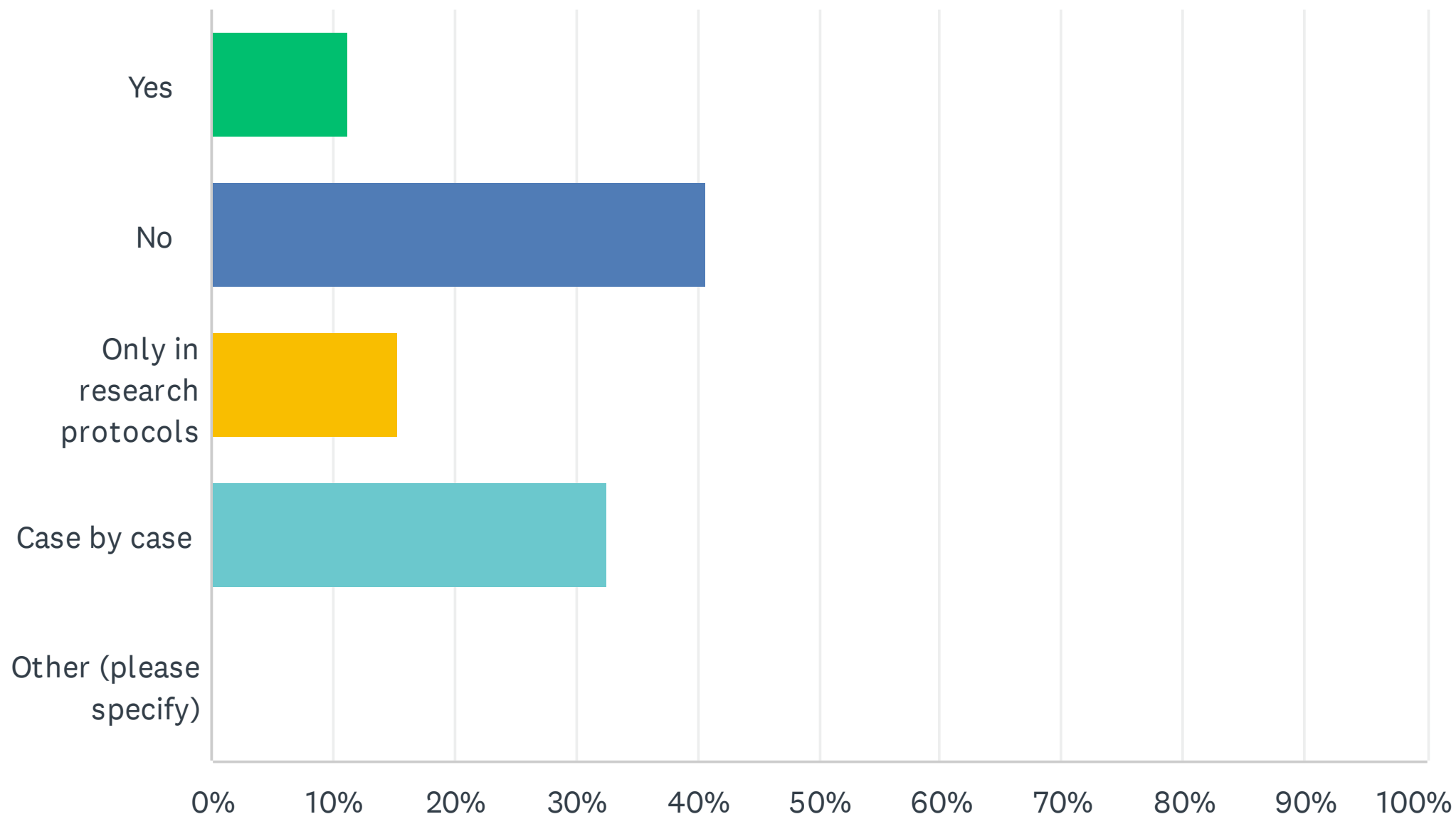

ANSWER CHOICES	RESPONSES	
Yes	11.22%	11
No	40.82%	40
Only in research protocols	15.31%	15
Case by case	32.65%	32
Other (please specify)	0.00%	0
TOTAL		98

Q6 Which non-invasive tests (NITs) do you use for routine assessment of PHT (select all that apply):

Answered: 98 Skipped: 16

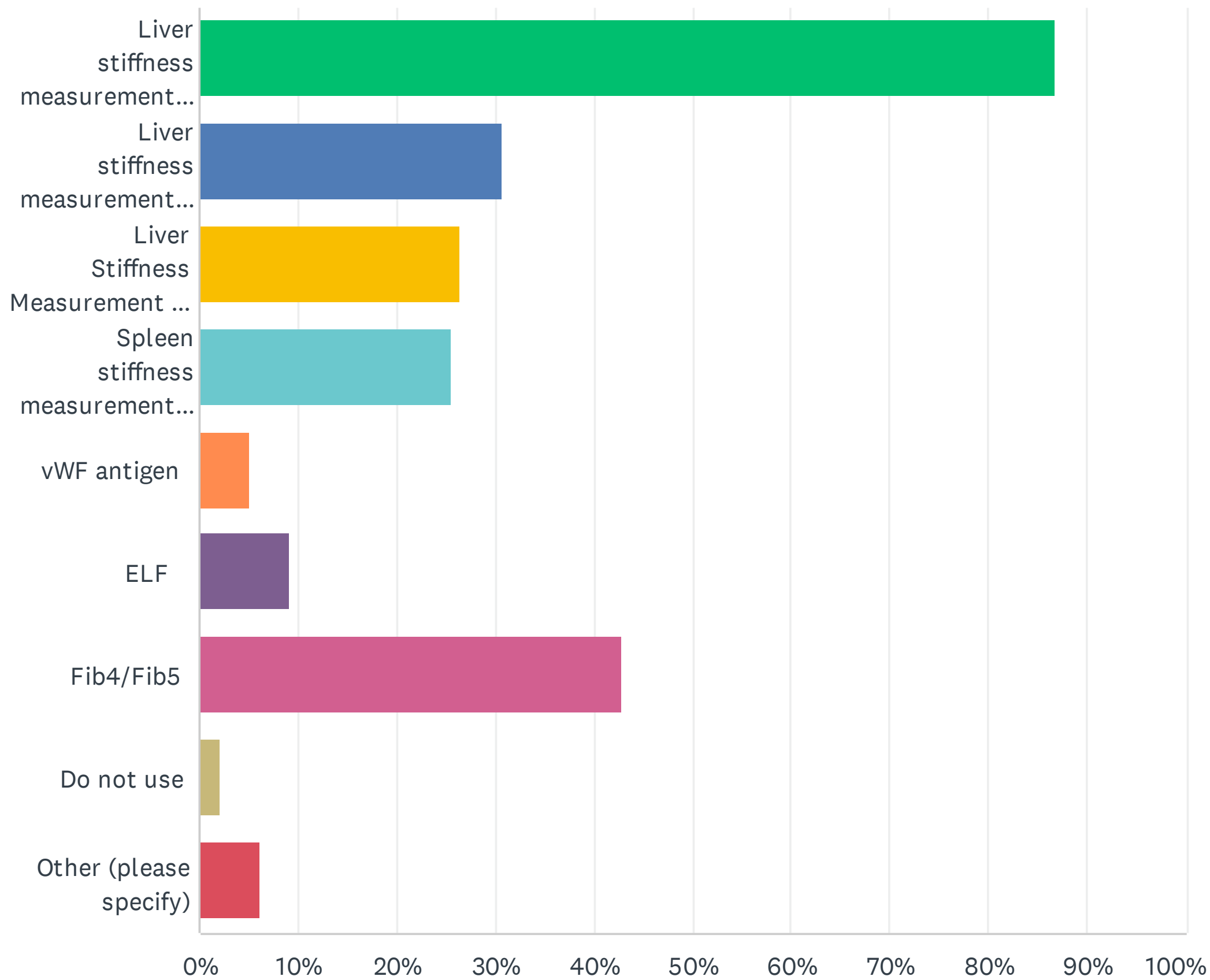

ANSWER CHOICES	RESPONSES	
Liver stiffness measurement (LSM)- Transient elastography	86.73%	85
Liver stiffness measurement –ultrasound based	30.61%	30
Liver Stiffness Measurement – MRE based	26.53%	26
Spleen stiffness measurement (SSM)	25.51%	25
vWF antigen	5.10%	5
ELF	9.18%	9
Fib4/Fib5	42.86%	42
Do not use	2.04%	2
Other (please specify)	6.12%	6
Total Respondents: 98		

Q7 Which is your preferred NSBB?

Answered: 98 Skipped: 16

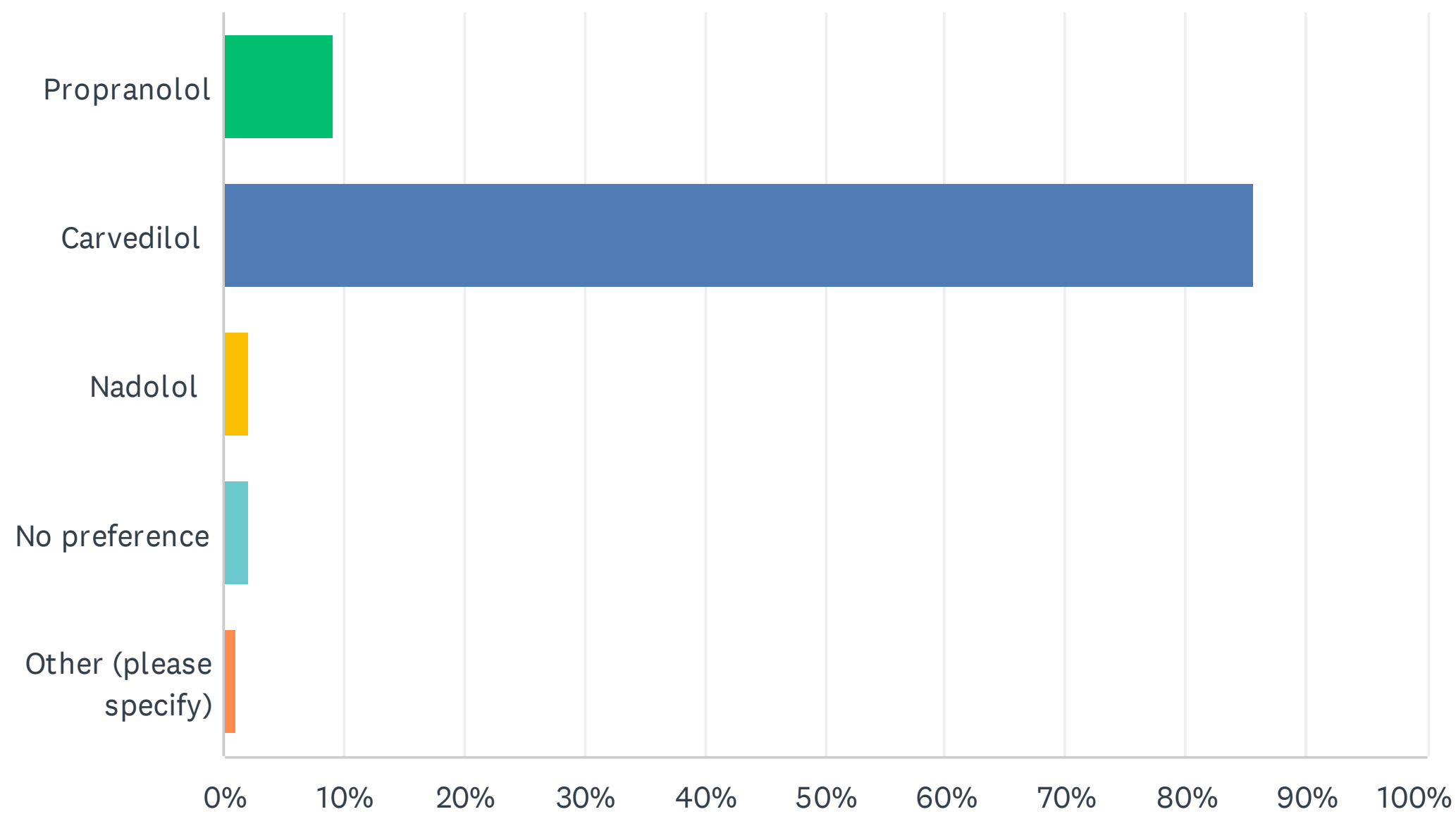

ANSWER CHOICES	RESPONSES	
Propranolol	9.18%	9
Carvedilol	85.71%	84
Nadolol	2.04%	2
No preference	2.04%	2
Other (please specify)	1.02%	1
TOTAL		98

Q8 For which indications do you routinely prescribe NSBB therapy for patients with cirrhosis? (select all that apply)

Answered: 98 Skipped: 16

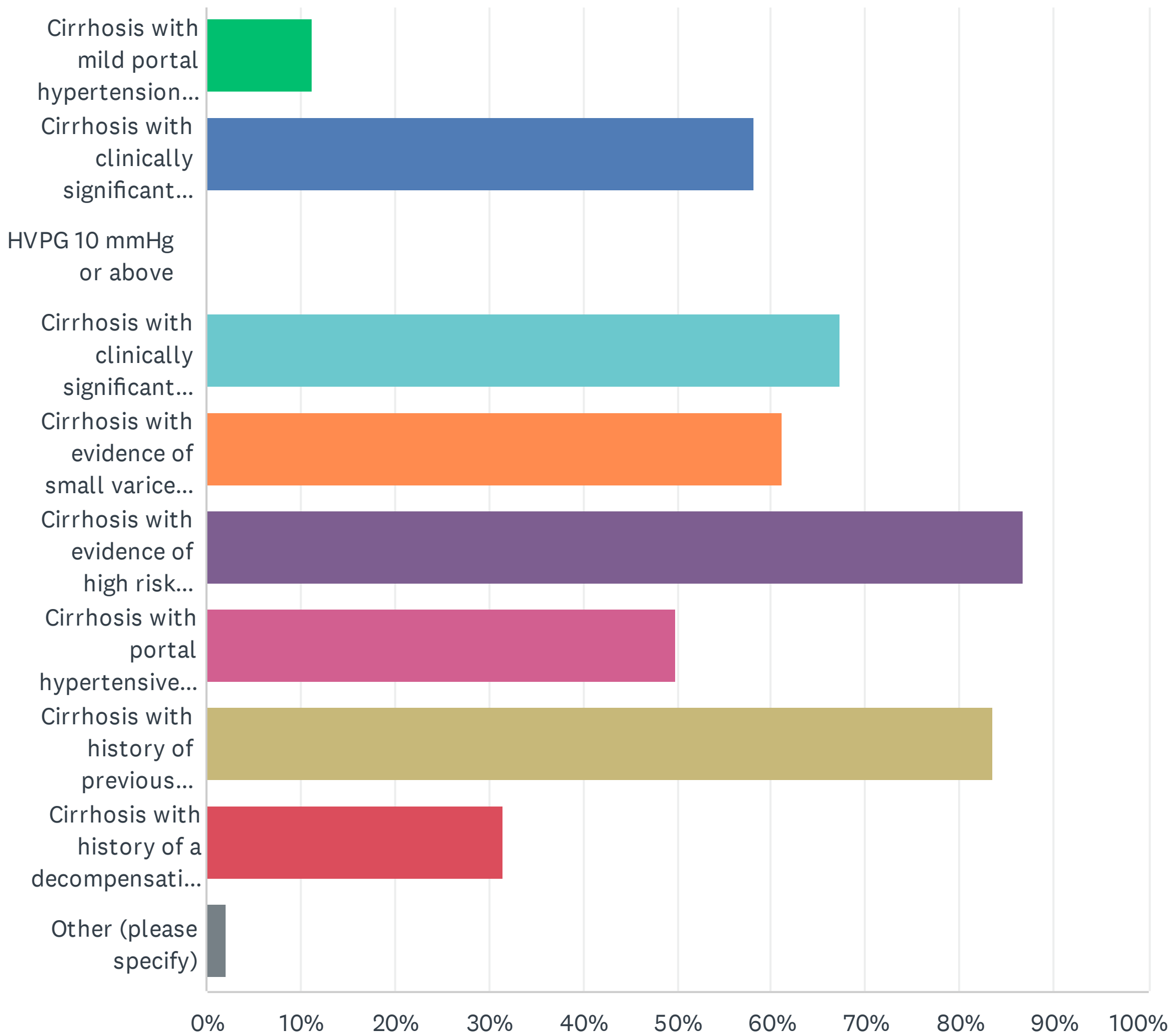

ANSWER CHOICES	RESPONSES	
Cirrhosis with mild portal hypertension based upon HVPG 6-10 mmHg	11.22%	11
Cirrhosis with clinically significant portal hypertension based upon HVPG 10 mmHg or above	58.16%	57
HVPG 10 mmHg or above	0.00%	0
Cirrhosis with clinically significant portal hypertension based on non-invasive testing	67.35%	66
Cirrhosis with evidence of small varices on endoscopy	61.22%	60
Cirrhosis with evidence of high risk varices on endoscopy	86.73%	85
Cirrhosis with portal hypertensive gastropathy without varices on endoscopy	50.00%	49
Cirrhosis with history of previous variceal bleeding	83.67%	82
Cirrhosis with history of a decompensation other than variceal bleeding (ascites or hepatic encephalopathy)	31.63%	31
Other (please specify)	2.04%	2
Total Respondents: 98		

Q9 Do you recommend use of NSBB in patients with cirrhosis regardless of screening endoscopy/size of varices in any of the following scenarios? (select all that apply)

Answered: 97 Skipped: 17

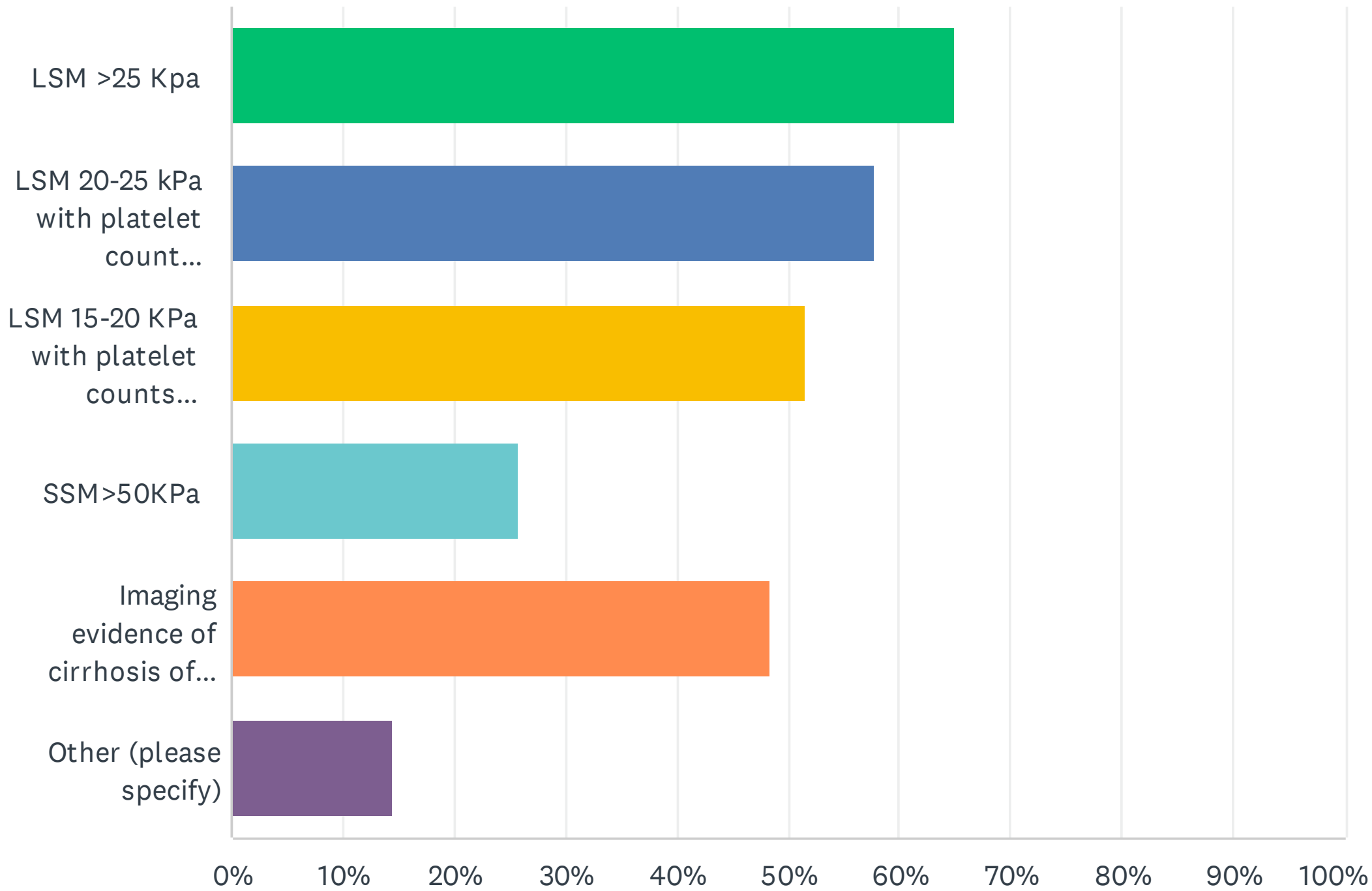

ANSWER CHOICES	RESPONSES	
LSM >25 Kpa	64.95%	63
LSM 20-25 kPa with platelet count <150,000/cm	57.73%	56
LSM 15-20 KPa with platelet counts <110,000/cm	51.55%	50
SSM>50KPa	25.77%	25
Imaging evidence of cirrhosis of liver with collaterals	48.45%	47
Other (please specify)	14.43%	14
Total Respondents: 97		

Q10 How do you assess response to NSBB?

Answered: 98 Skipped: 16

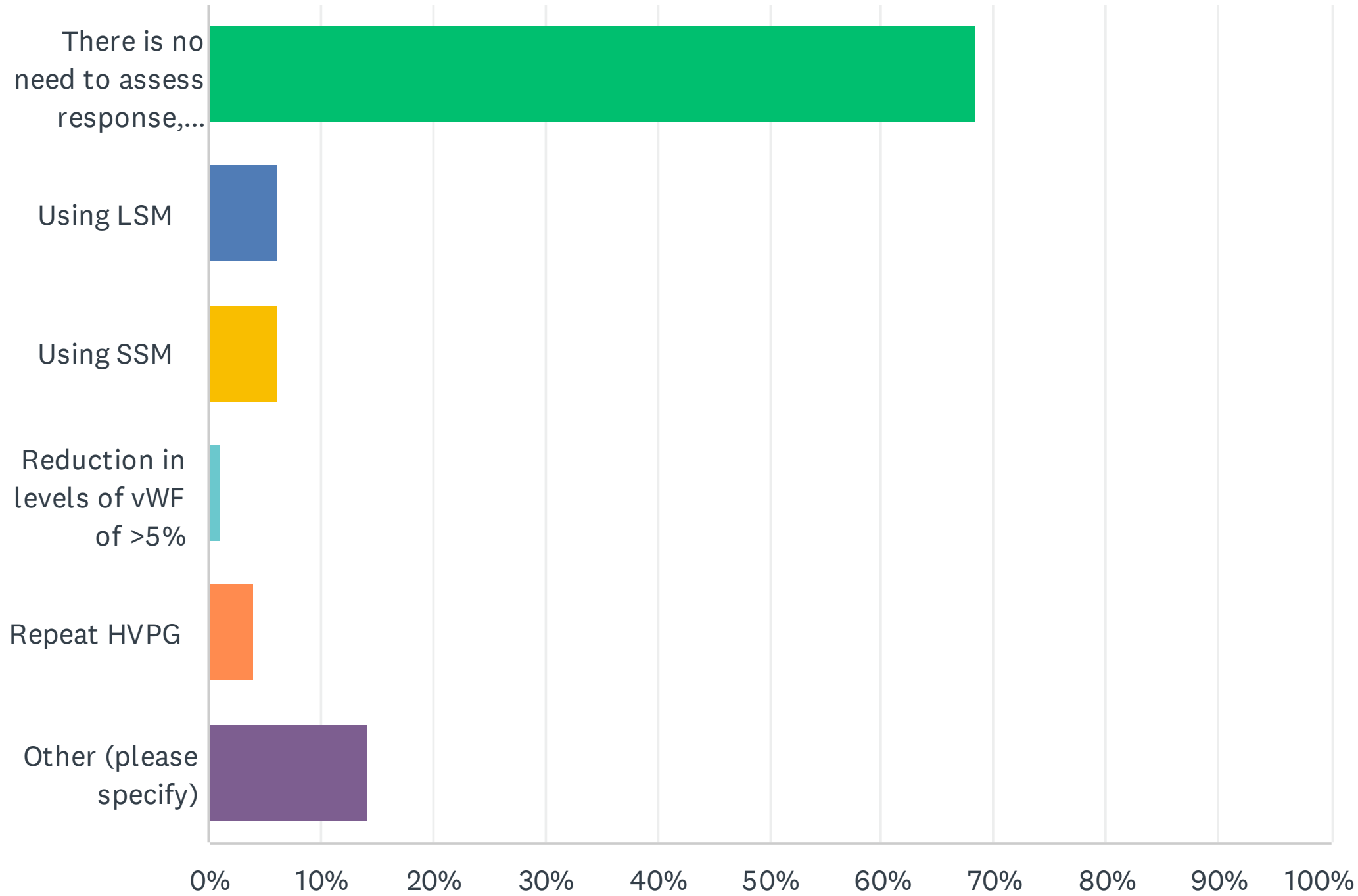

ANSWER CHOICES	RESPONSES	
There is no need to assess response, compliance and tolerance can be assessed via heart rate to 55-60 bpm	68.37%	67
Using LSM	6.12%	6
Using SSM	6.12%	6
Reduction in levels of vWF of >5%	1.02%	1
Repeat HVPG	4.08%	4
Other (please specify)	14.29%	14
TOTAL		98

Q11 What criteria do you use when determining whether a patient is intolerant to NSBB? (select all that apply)

Answered: 98 Skipped: 16

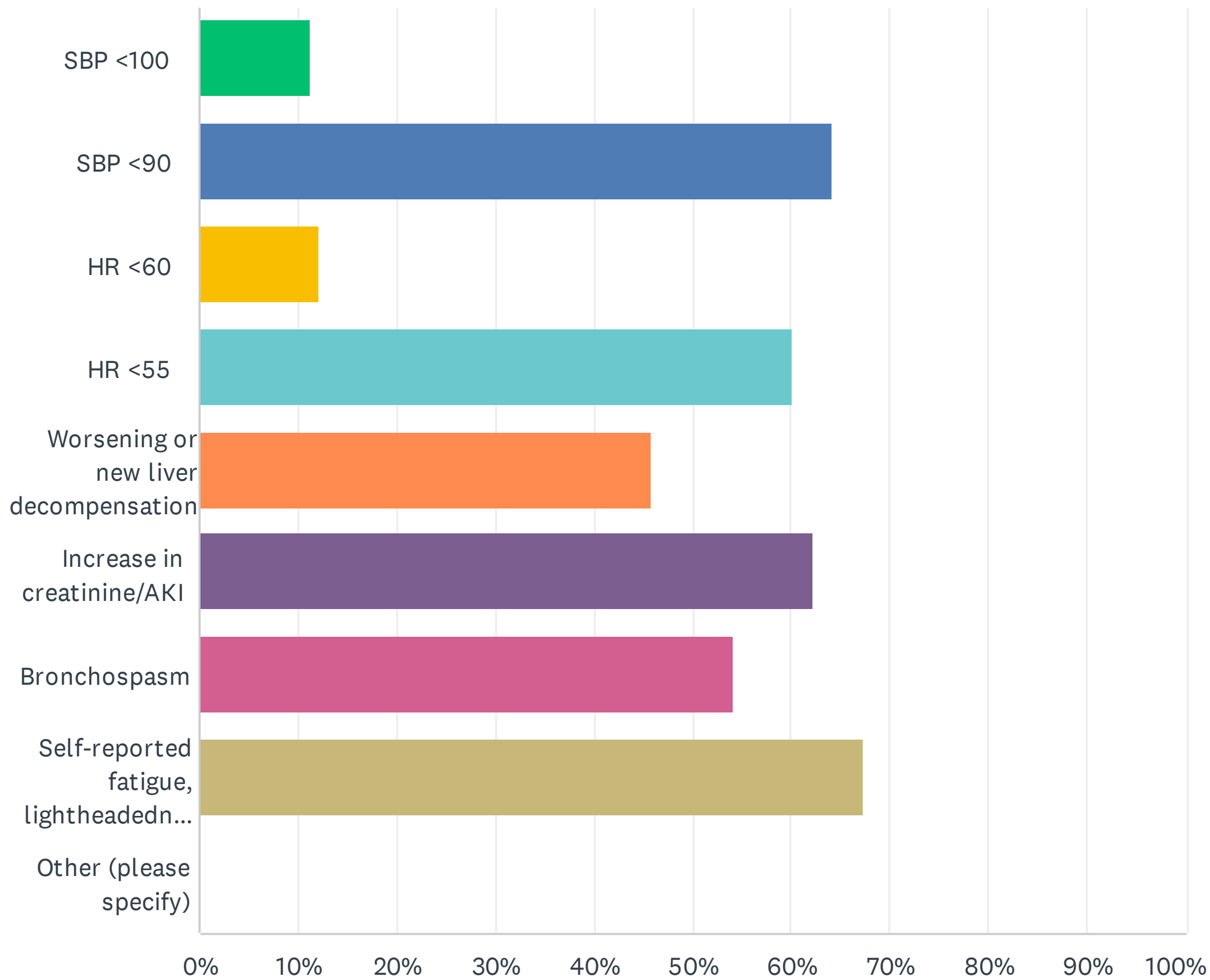

ANSWER CHOICES	RESPONSES	
SBP <100	11.22%	11
SBP <90	64.29%	63
HR <60	12.24%	12
HR <55	60.20%	59
Worsening or new liver decompensation	45.92%	45
Increase in creatinine/AKI	62.24%	61
Bronchospasm	54.08%	53
Self-reported fatigue, lightheadedness, and/or giddiness	67.35%	66
Other (please specify)	0.00%	0
Total Respondents: 98		

Q12 How do you approach encouraging long-term adherence with the prescribed NSBB? (Select all that apply)

Answered: 97 Skipped: 17

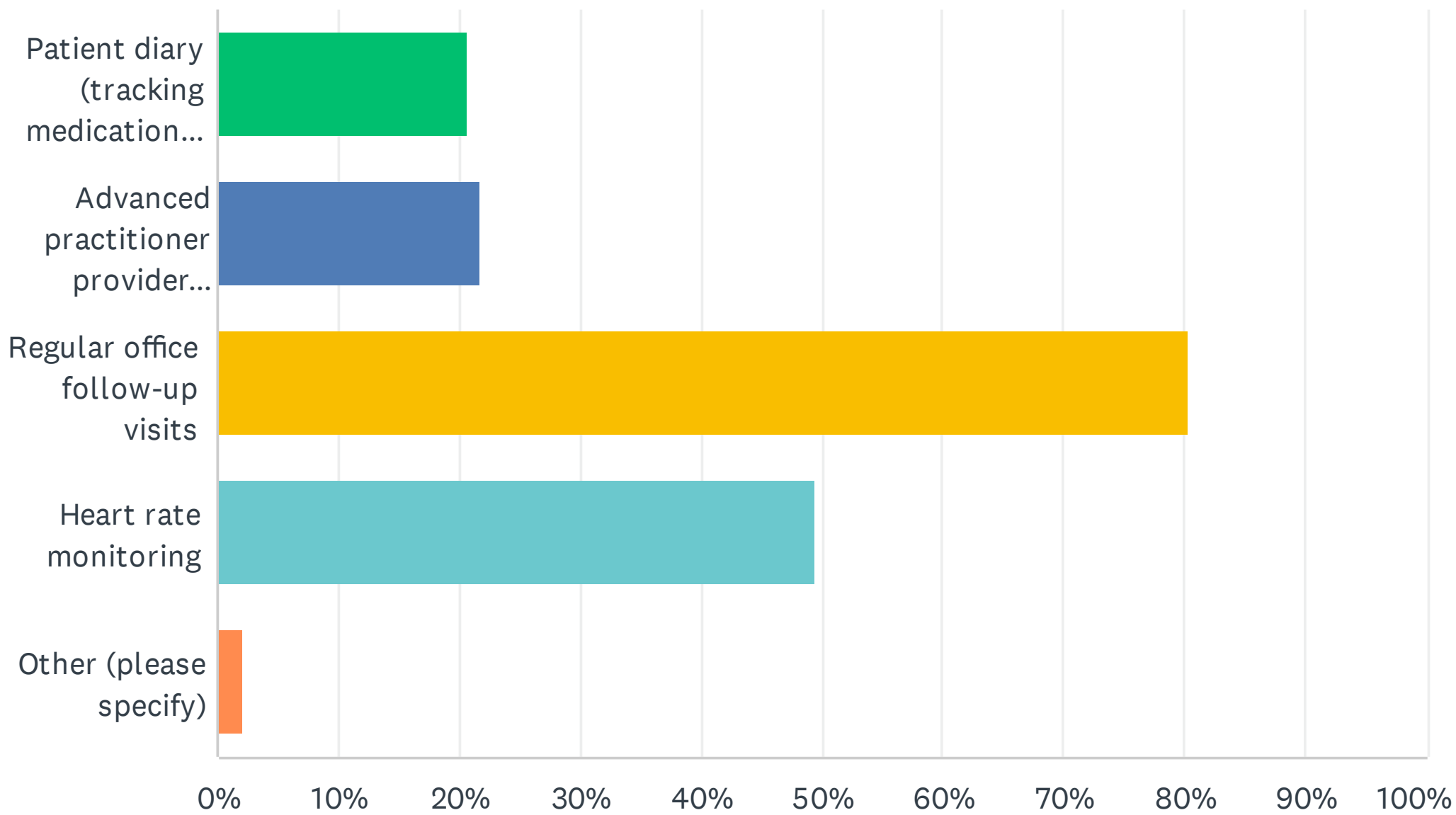

ANSWER CHOICES	RESPONSES	
Patient diary (tracking medication adherence)	20.62%	20
Advanced practitioner provider follow-up	21.65%	21
Regular office follow-up visits	80.41%	78
Heart rate monitoring	49.48%	48
Other (please specify)	2.06%	2
Total Respondents: 97		

Q13 Do you use NSBB in patients with cirrhosis and high-risk varices who have ascites for primary prophylaxis? (Select all that apply)

Answered: 98 Skipped: 16

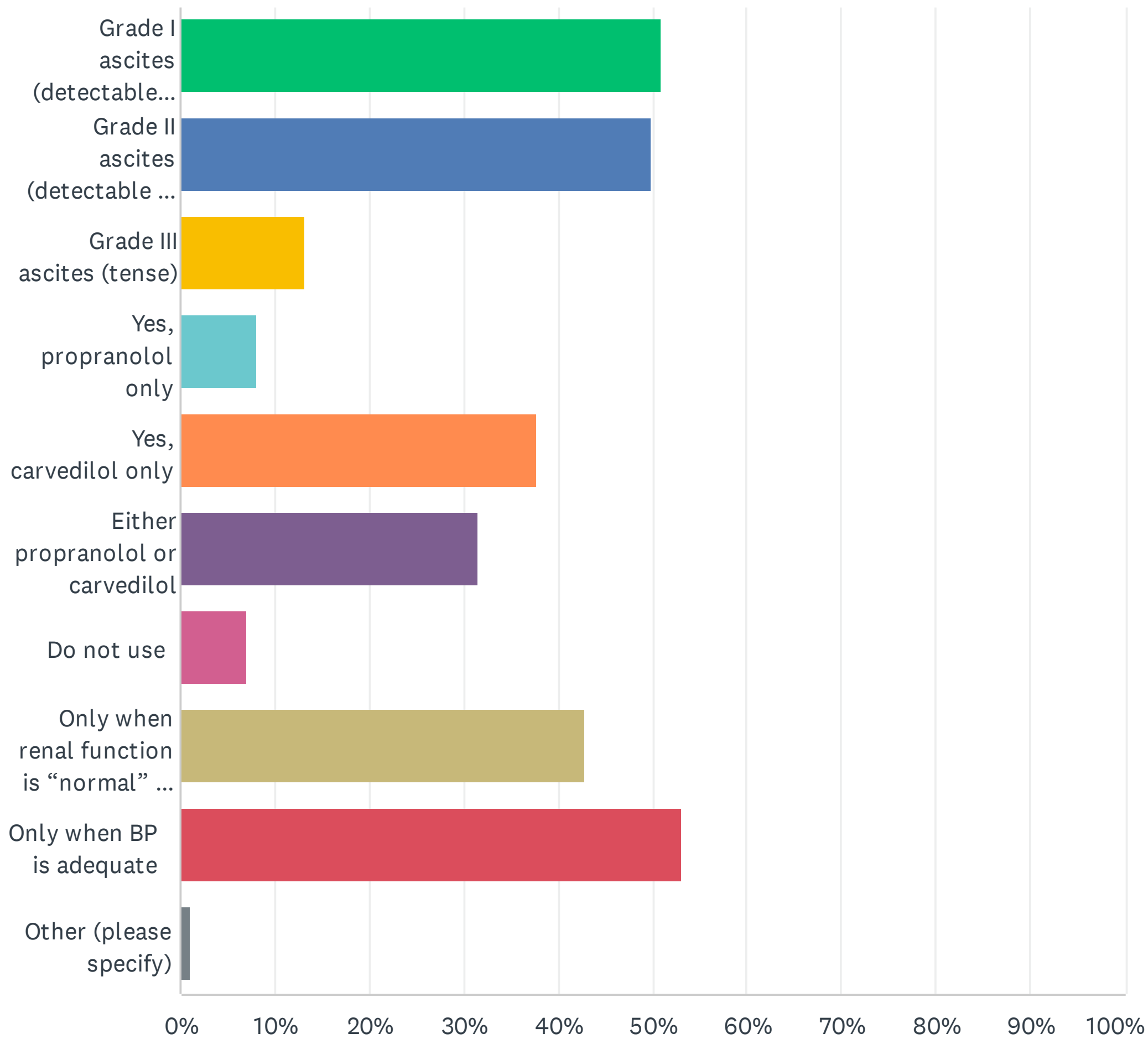

ANSWER CHOICES	RESPONSES	
Grade I ascites (detectable only on imaging) with creatinine >1.0 mg/dl	51.02%	50
Grade II ascites (detectable by physical exam)	50.00%	49
Grade III ascites (tense)	13.27%	13
Yes, propranolol only	8.16%	8
Yes, carvedilol only	37.76%	37
Either propranolol or carvedilol	31.63%	31
Do not use	7.14%	7
Only when renal function is “normal” at baseline	42.86%	42
Only when BP is adequate	53.06%	52
Other (please specify)	1.02%	1
Total Respondents: 98		

Q14 In patients with large, high risk, gastric varices, for primary prophylaxis, which of the following do you use? (Select all that apply)

Answered: 98 Skipped: 16

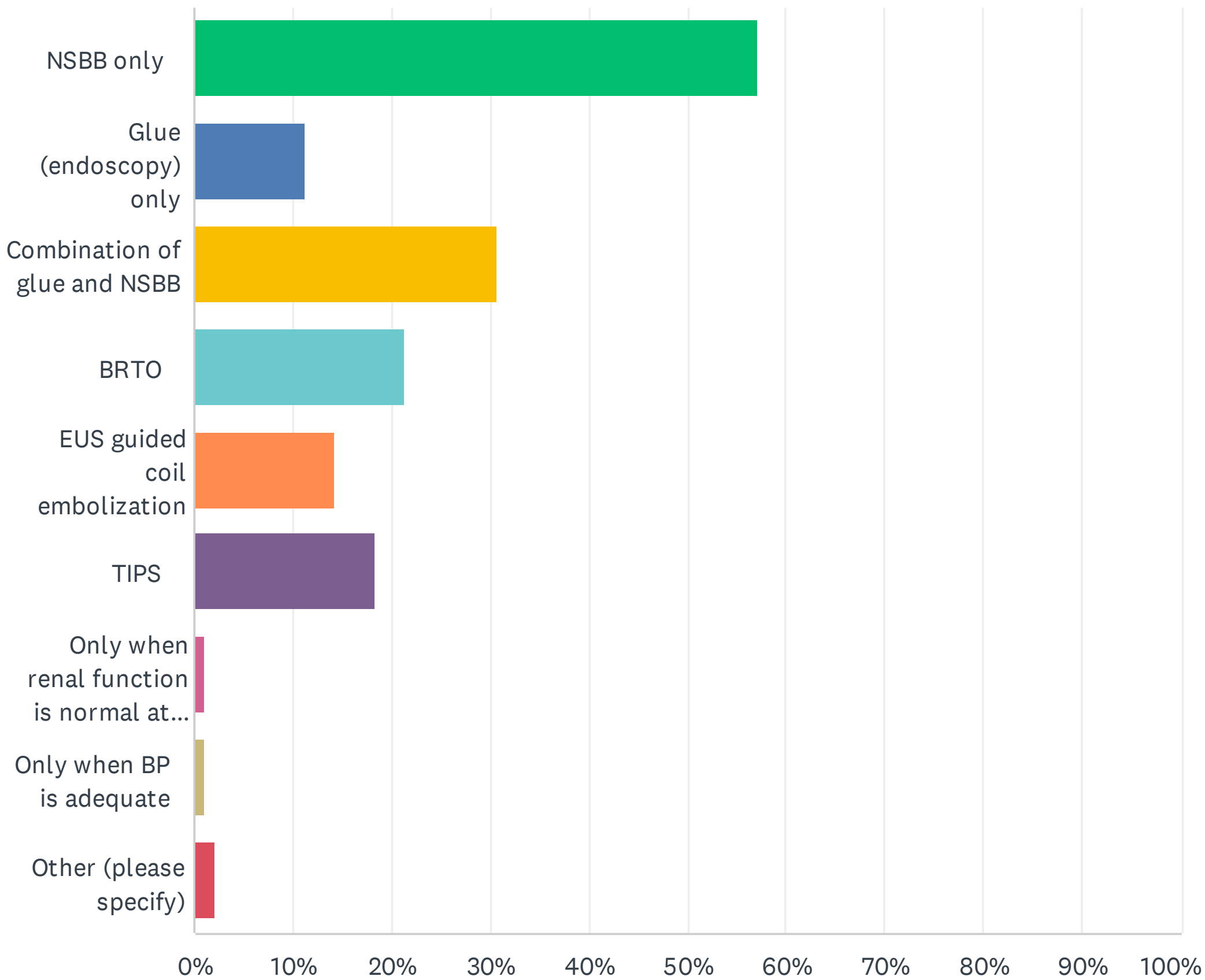

ANSWER CHOICES	RESPONSES	
NSBB only	57.14%	56
Glue (endoscopy) only	11.22%	11
Combination of glue and NSBB	30.61%	30
BRTO	21.43%	21
EUS guided coil embolization	14.29%	14
TIPS	18.37%	18
Only when renal function is normal at baseline	1.02%	1
Only when BP is adequate	1.02%	1
Other (please specify)	2.04%	2
Total Respondents: 98		

Q15 In patients with gastric varices who have bled, for secondary prophylaxis, do you use any of the following? (Select all that apply)

Answered: 98 Skipped: 16

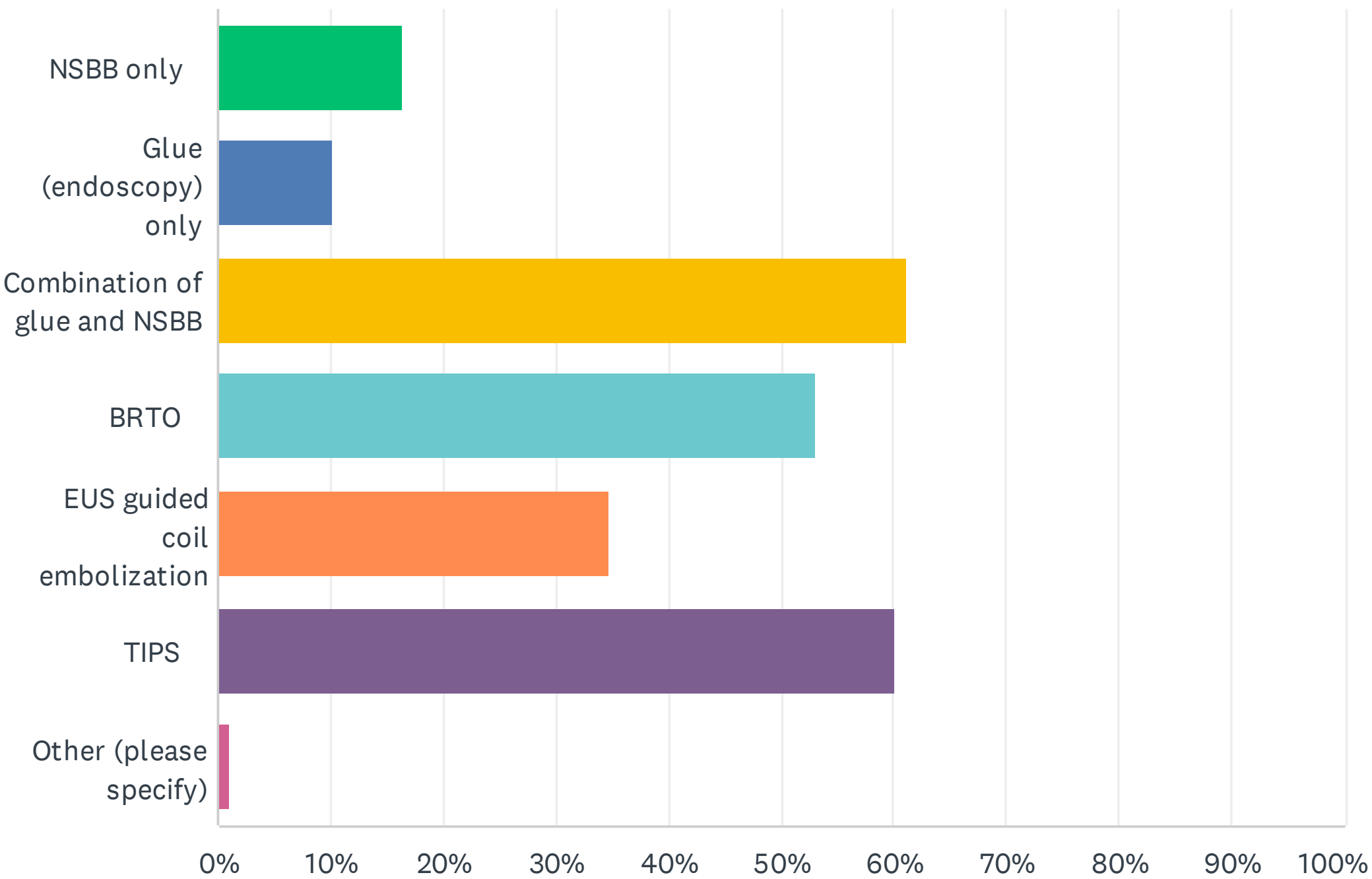

ANSWER CHOICES	RESPONSES	
NSBB only	16.33%	16
Glue (endoscopy) only	10.20%	10
Combination of glue and NSBB	61.22%	60
BRTO	53.06%	52
EUS guided coil embolization	34.69%	34
TIPS	60.20%	59
Other (please specify)	1.02%	1
Total Respondents: 98		

Q16 In patients with severe portal hypertensive gastropathy without evidence of active GI bleeding, do you use any of the following for primary prophylaxis? (Select all that apply)

Answered: 98 Skipped: 16

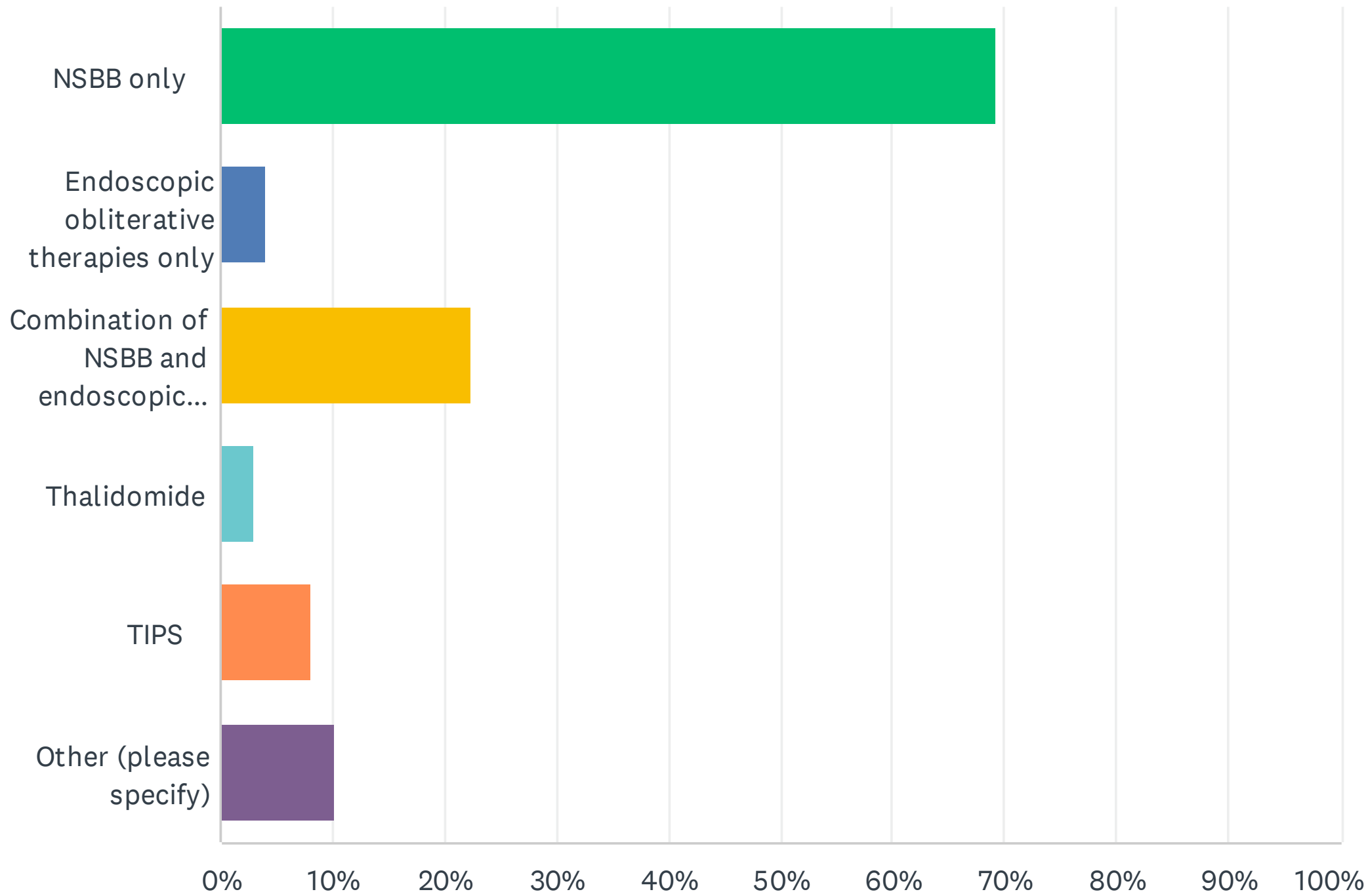

ANSWER CHOICES	RESPONSES	
NSBB only	69.39%	68
Endoscopic obliterative therapies only	4.08%	4
Combination of NSBB and endoscopic obliterative therapies	22.45%	22
Thalidomide	3.06%	3
TIPS	8.16%	8
Other (please specify)	10.20%	10
Total Respondents: 98		

Q17 In patients with severe portal hypertensive gastropathy with evidence of chronic GI bleeding (chronic blood loss, anemia,), do you use any of the following? (Select all that apply)

Answered: 97 Skipped: 17

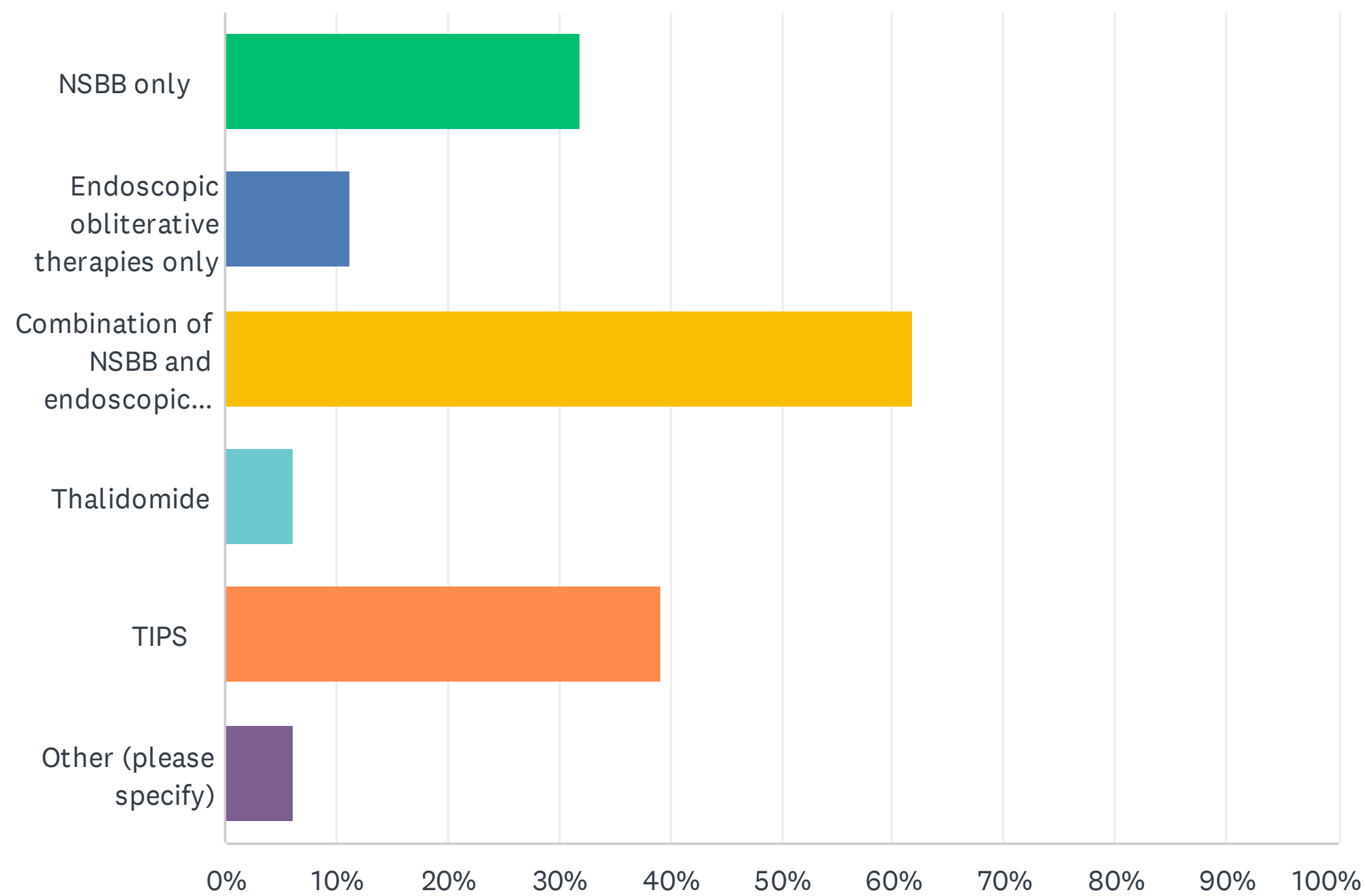

ANSWER CHOICES	RESPONSES	
NSBB only	31.96%	31
Endoscopic obliterative therapies only	11.34%	11
Combination of NSBB and endoscopic obliterative therapies	61.86%	60
Thalidomide	6.19%	6
TIPS	39.18%	38
Other (please specify)	6.19%	6
Total Respondents: 97		

Q18 Do you use NSBB in cirrhosis patients with AKI (creatinine > 2 mg/dL)? (Select all that apply)

Answered: 88 Skipped: 26

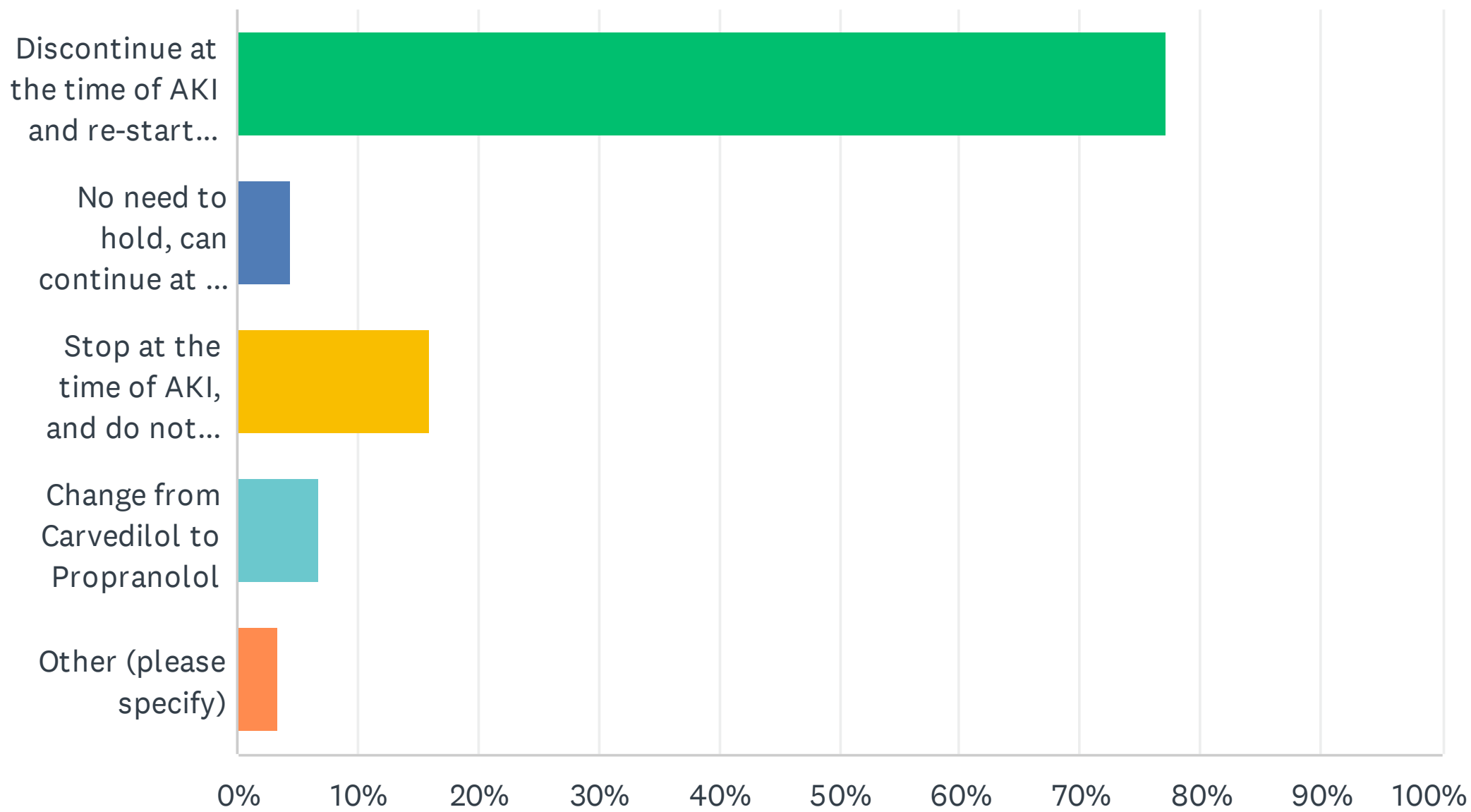

ANSWER CHOICES	RESPONSES	
Discontinue at the time of AKI and re-start when recovered	77.27%	68
No need to hold, can continue at the time of AKI	4.55%	4
Stop at the time of AKI, and do not re-start even after recovery	15.91%	14
Change from Carvedilol to Propranolol	6.82%	6
Other (please specify)	3.41%	3
Total Respondents: 88		

Q19 In patients for variceal bleed prophylaxis where despite an adequate dosage (12.5 mg b.i.d of carvedilol or 160 mg of propranolol), a HR of 55-60 bpm cannot be achieved while at the same time maintaining systolic pressure (Select all that apply)

Answered: 88 Skipped: 26

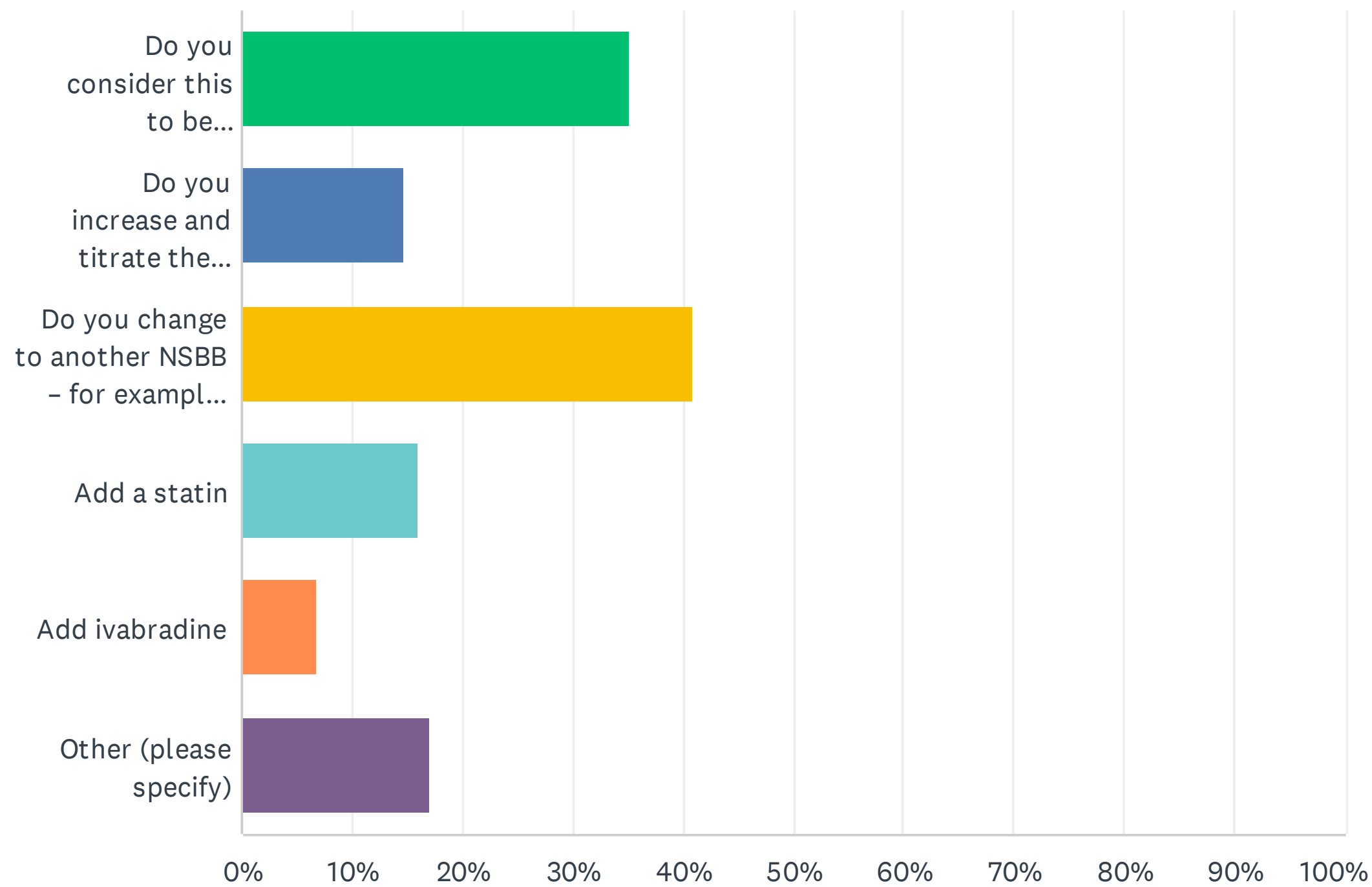

ANSWER CHOICES	RESPONSES	
Do you consider this to be intolerance (or non-response) to NSBB	35.23%	31
Do you increase and titrate the dose to achieve target heart rate of 55-60 bpm	14.77%	13
Do you change to another NSBB – for example, from propranolol to carvedilol	40.91%	36
Add a statin	15.91%	14
Add ivabradine	6.82%	6
Other (please specify)	17.05%	15
Total Respondents: 88		

Q20 In patients with cirrhosis, ascites, and varices on EGD, for primary prophylaxis of variceal bleed in high-risk varices, if the MAP goes <70 mmHg on a NSBB, do you consider any of the following (select all that apply):

Answered: 88 Skipped: 26

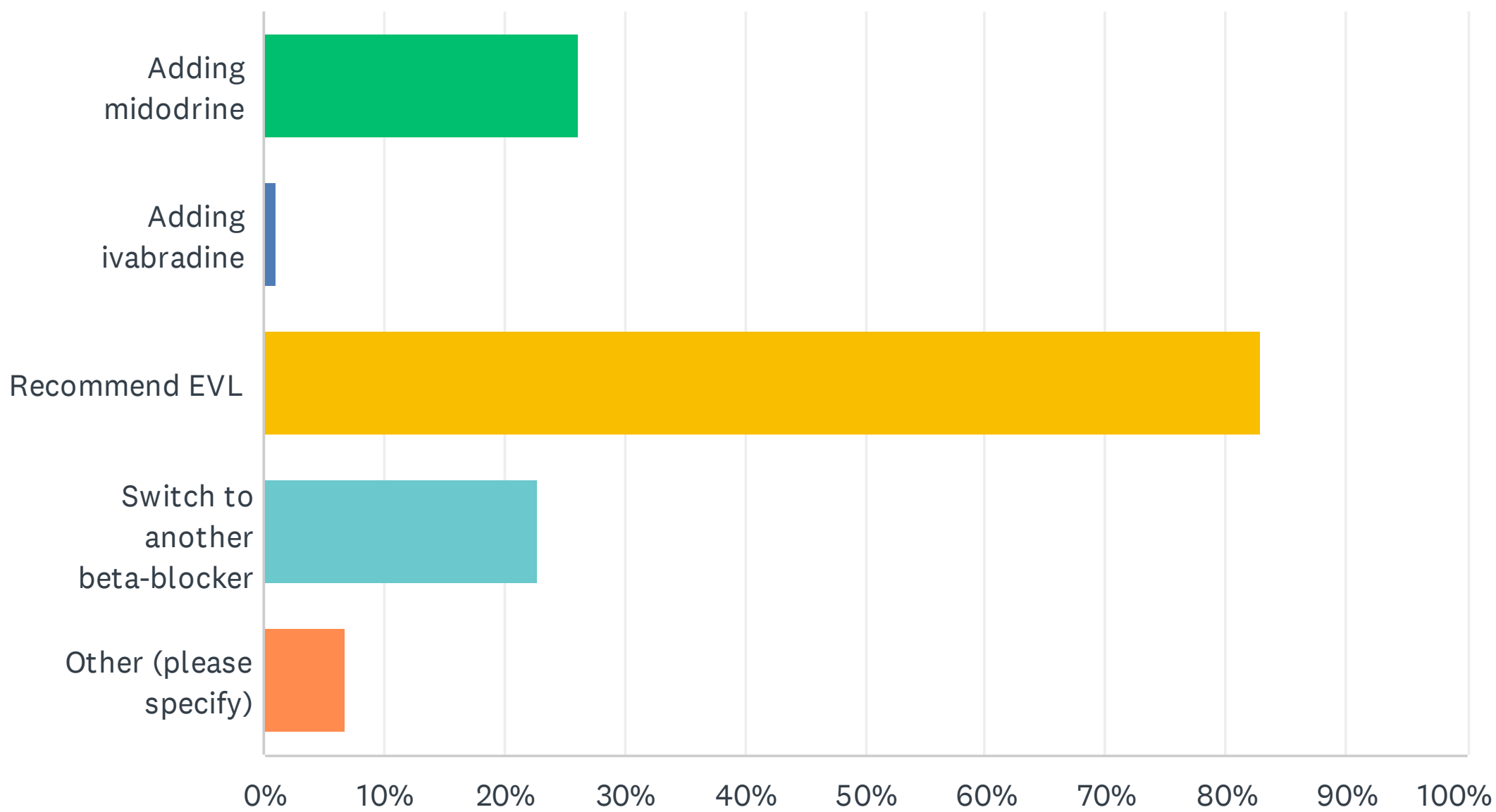

ANSWER CHOICES	RESPONSES	
Adding midodrine	26.14%	23
Adding ivabradine	1.14%	1
Recommend EVL	82.95%	73
Switch to another beta-blocker	22.73%	20
Other (please specify)	6.82%	6
Total Respondents: 88		

Q21 In patients with cirrhosis and HCC with portal vein thrombosis requiring SBRT/TARE/TACE do you recommend NSBB in patients with any of the following:

Answered: 88 Skipped: 26

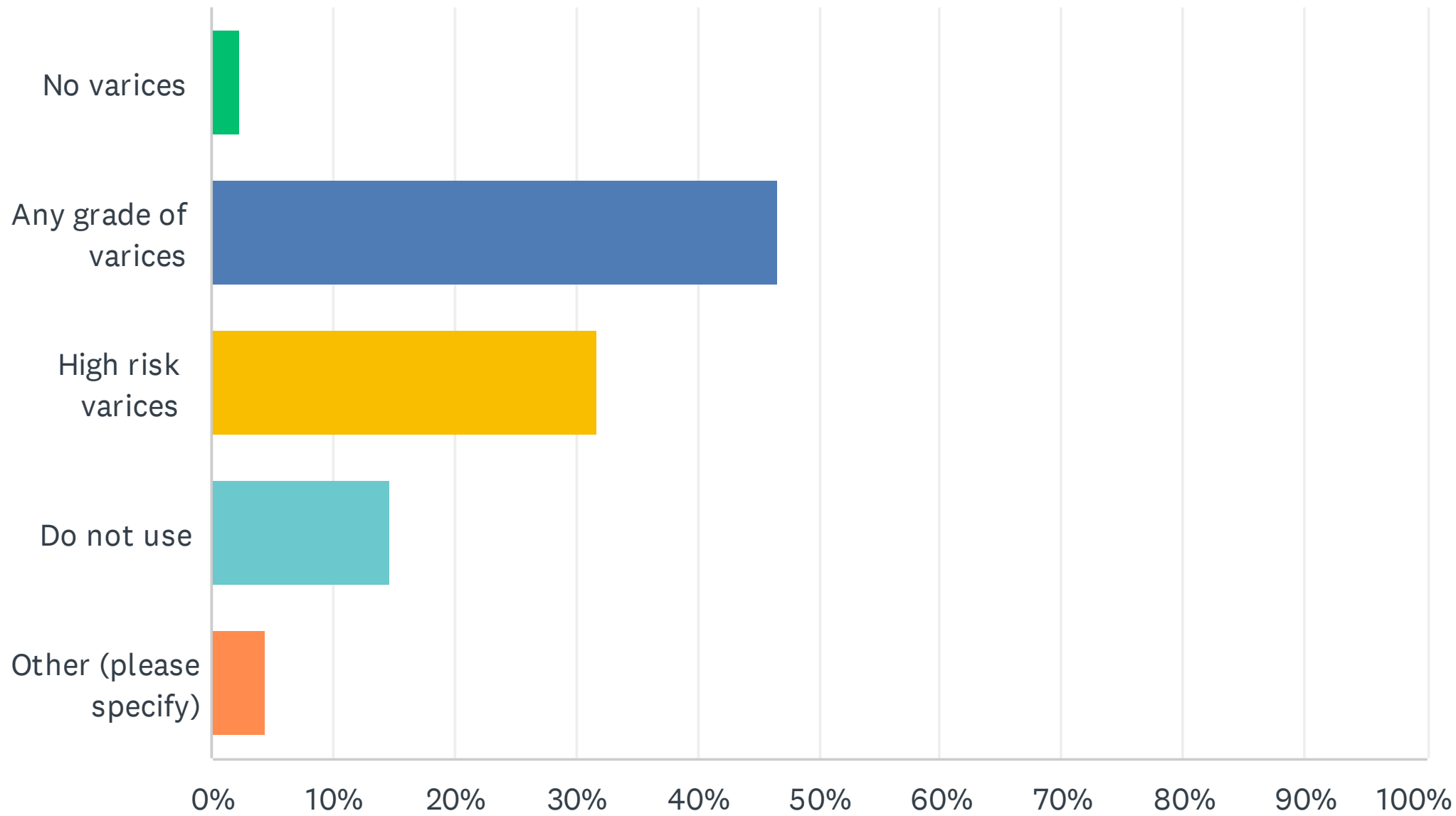

ANSWER CHOICES	RESPONSES	
No varices	2.27%	2
Any grade of varices	46.59%	41
High risk varices	31.82%	28
Do not use	14.77%	13
Other (please specify)	4.55%	4
TOTAL		88

Q22 In patients taking NSBB presenting with variceal hemorrhage, and a heart rate below 60 bpm and SBP < 90 mm Hg, do you use any of the following:

Answered: 89 Skipped: 25

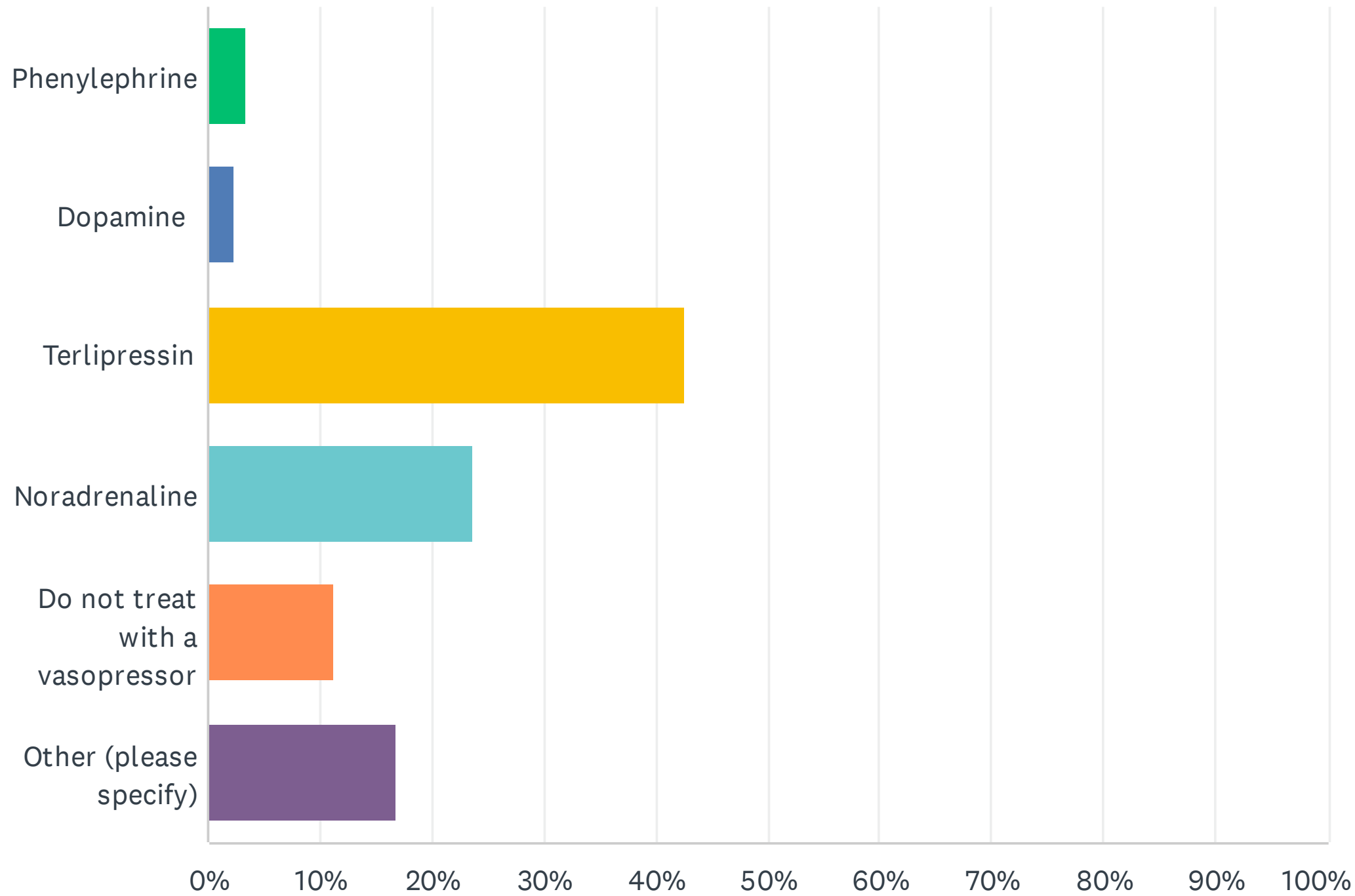

ANSWER CHOICES	RESPONSES	
Phenylephrine	3.37%	3
Dopamine	2.25%	2
Terlipressin	42.70%	38
Noradrenaline	23.60%	21
Do not treat with a vasopressor	11.24%	10
Other (please specify)	16.85%	15
TOTAL		89

Q23 In patients with portal hypertension complications undergoing TIPS placement for variceal bleeding, with HVPG reduction to less than 10 mmHg, do you start or continue the NSBB (select all that apply)

Answered: 88 Skipped: 26

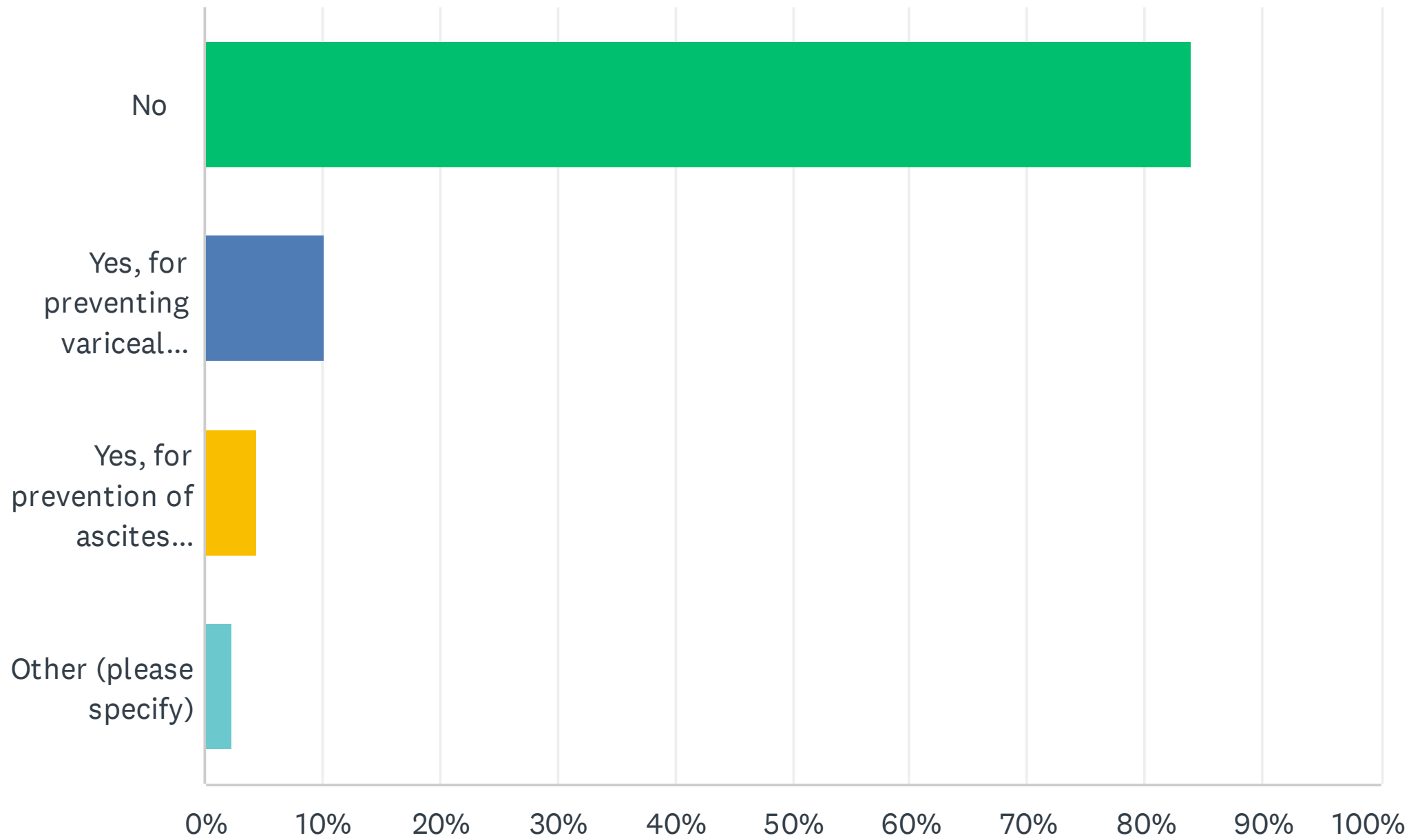

ANSWER CHOICES	RESPONSES	
No	84.09%	74
Yes, for preventing variceal recurrence	10.23%	9
Yes, for prevention of ascites progression	4.55%	4
Other (please specify)	2.27%	2
Total Respondents: 88		

Q24 In patients hospitalized with ACLF (of any grade) and ascites and MELD >20, with high-risk varices, what is your approach?

Answered: 89 Skipped: 25

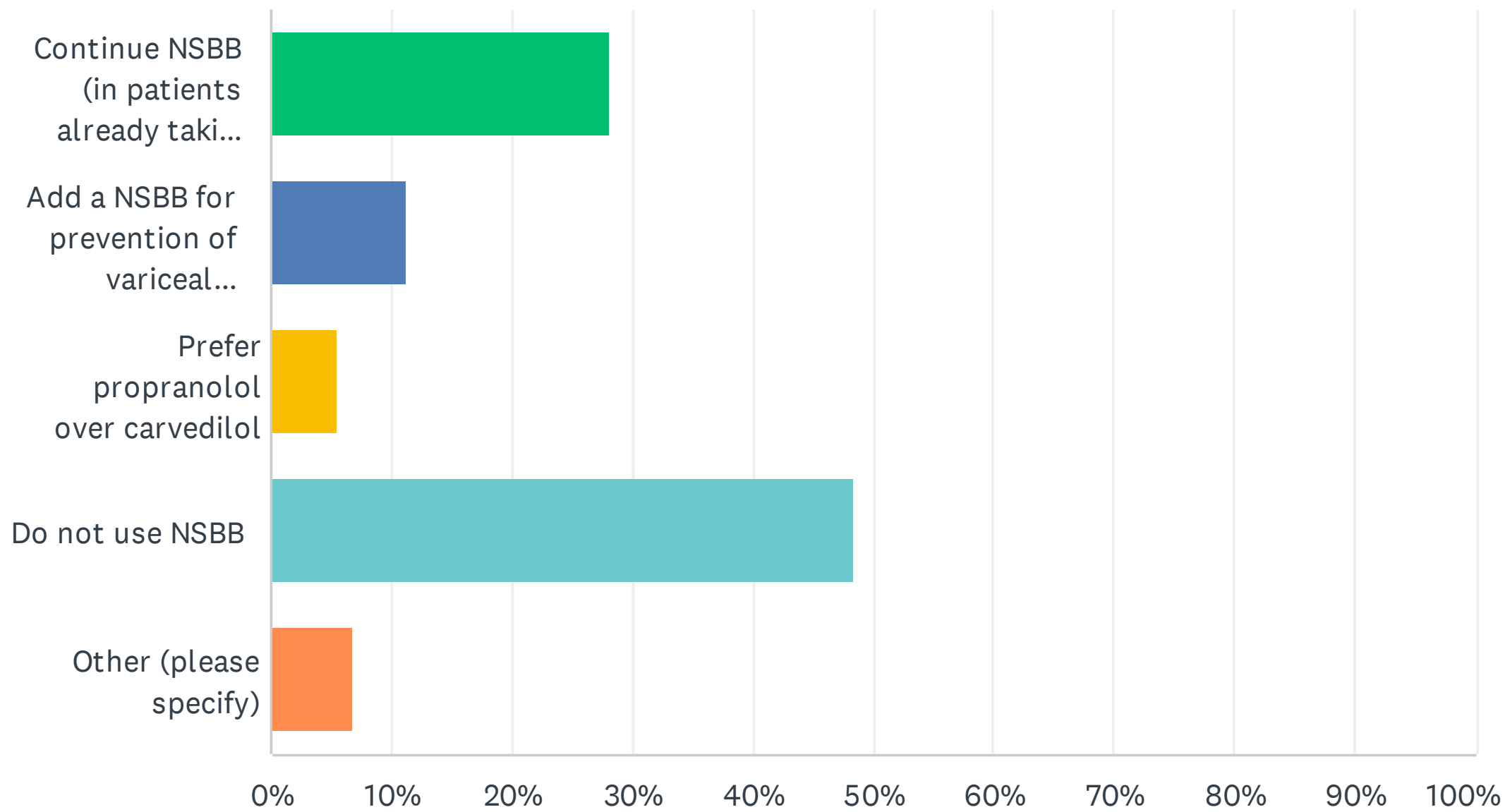

ANSWER CHOICES	RESPONSES	
Continue NSBB (in patients already taking NSBB)	28.09%	25
Add a NSBB for prevention of variceal bleeding	11.24%	10
Prefer propranolol over carvedilol	5.62%	5
Do not use NSBB	48.31%	43
Other (please specify)	6.74%	6
TOTAL		89

Q25 In patients with decompensated cirrhosis and MELD >20, high-risk varices and minimal ascites, which of the following do you recommend?

Answered: 89 Skipped: 25

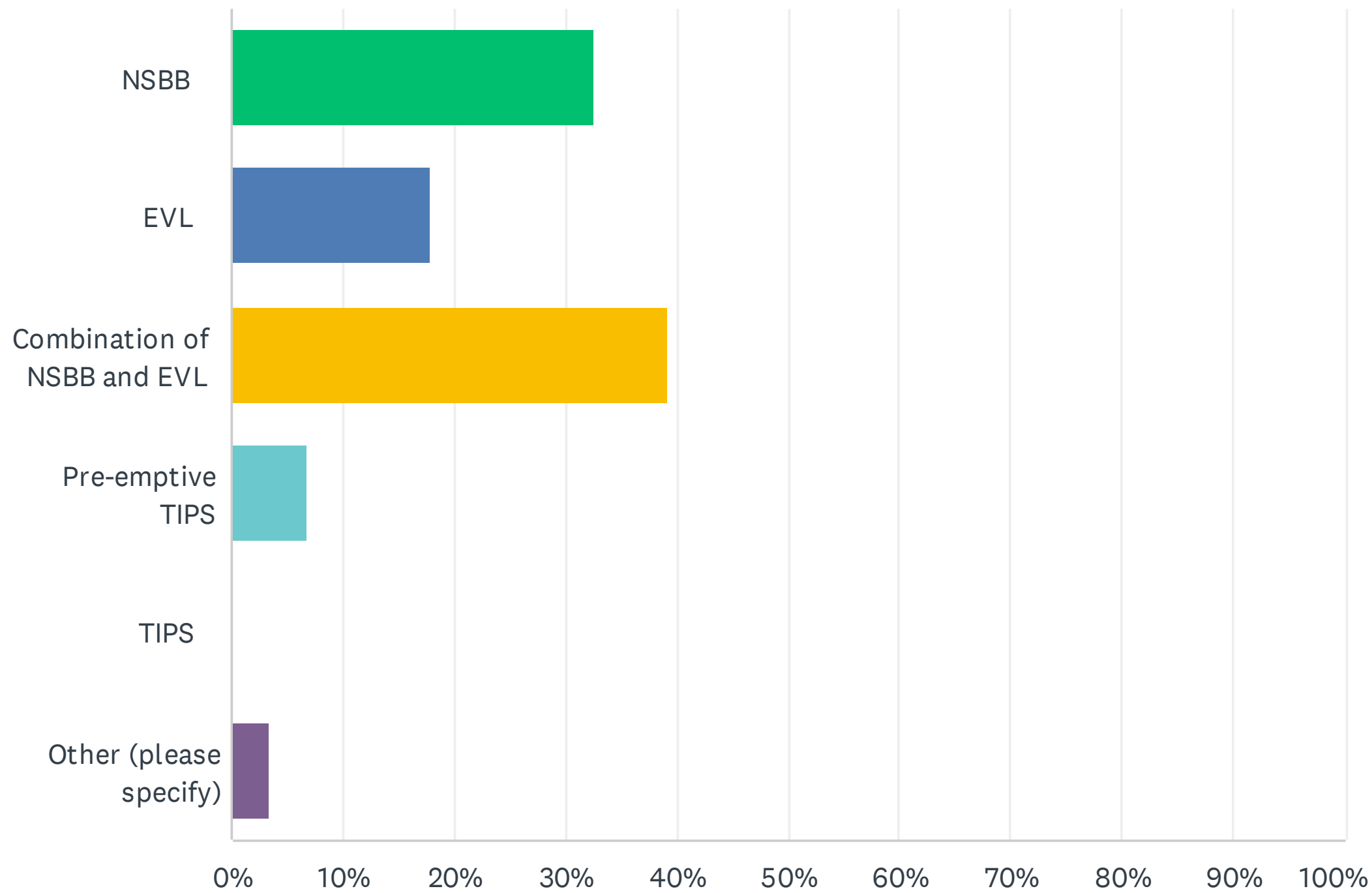

ANSWER CHOICES	RESPONSES	
NSBB	32.58%	29
EVL	17.98%	16
Combination of NSBB and EVL	39.33%	35
Pre-emptive TIPS	6.74%	6
TIPS	0.00%	0
Other (please specify)	3.37%	3
TOTAL		89

Q26 In patients with cirrhosis with high-risk varices (without previous bleeding) with a past h/o HE and a large spleno-renal shunt, which of the following do you recommend?

Answered: 88 Skipped: 26

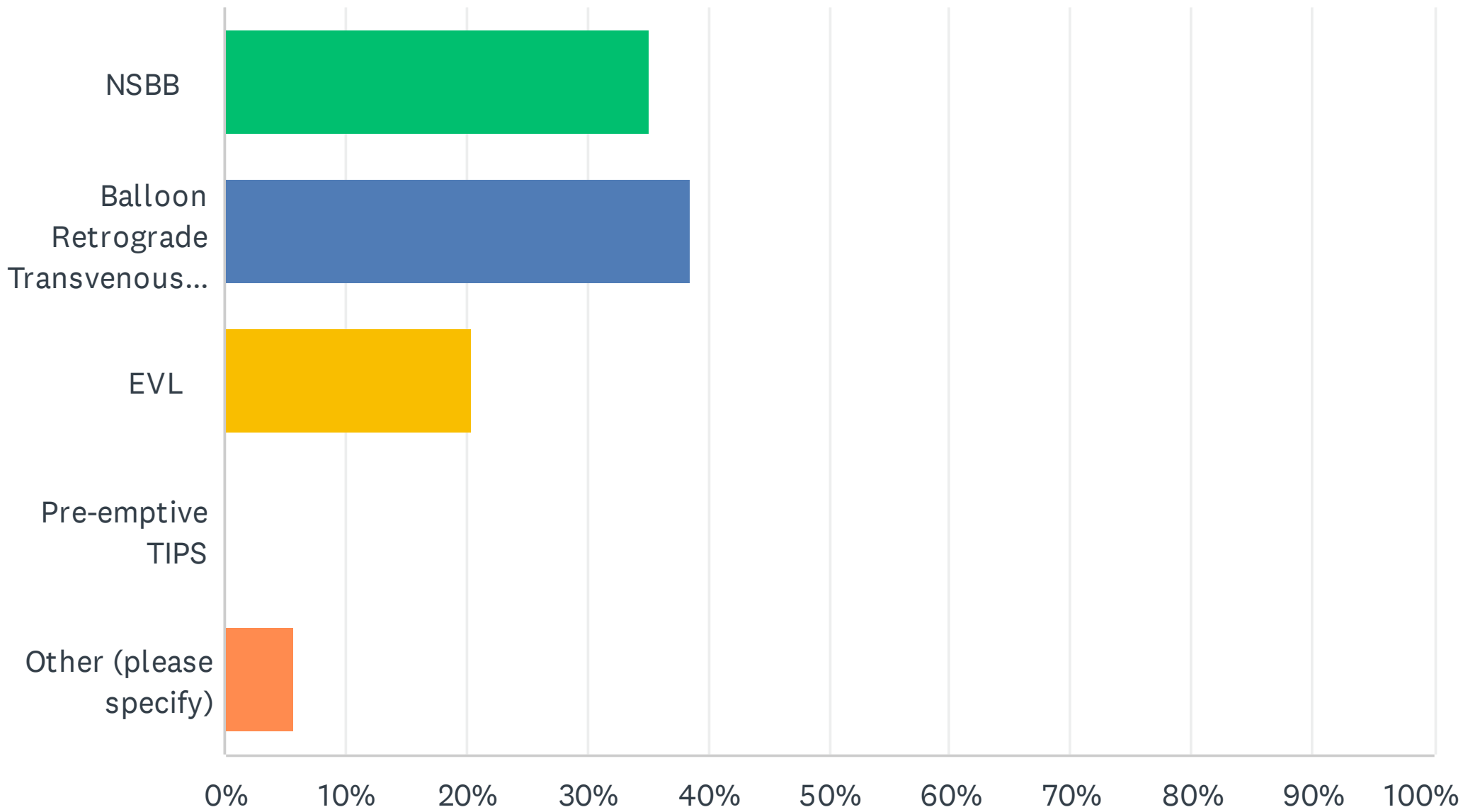

ANSWER CHOICES	RESPONSES	
NSBB	35.23%	31
Balloon Retrograde Transvenous Obliteration (BRTO)	38.64%	34
EVL	20.45%	18
Pre-emptive TIPS	0.00%	0
Other (please specify)	5.68%	5
TOTAL		88

Q27 Inpatients with alcohol associated severe hepatitis, with no varices, do you use (Select all that apply)

Answered: 88 Skipped: 26

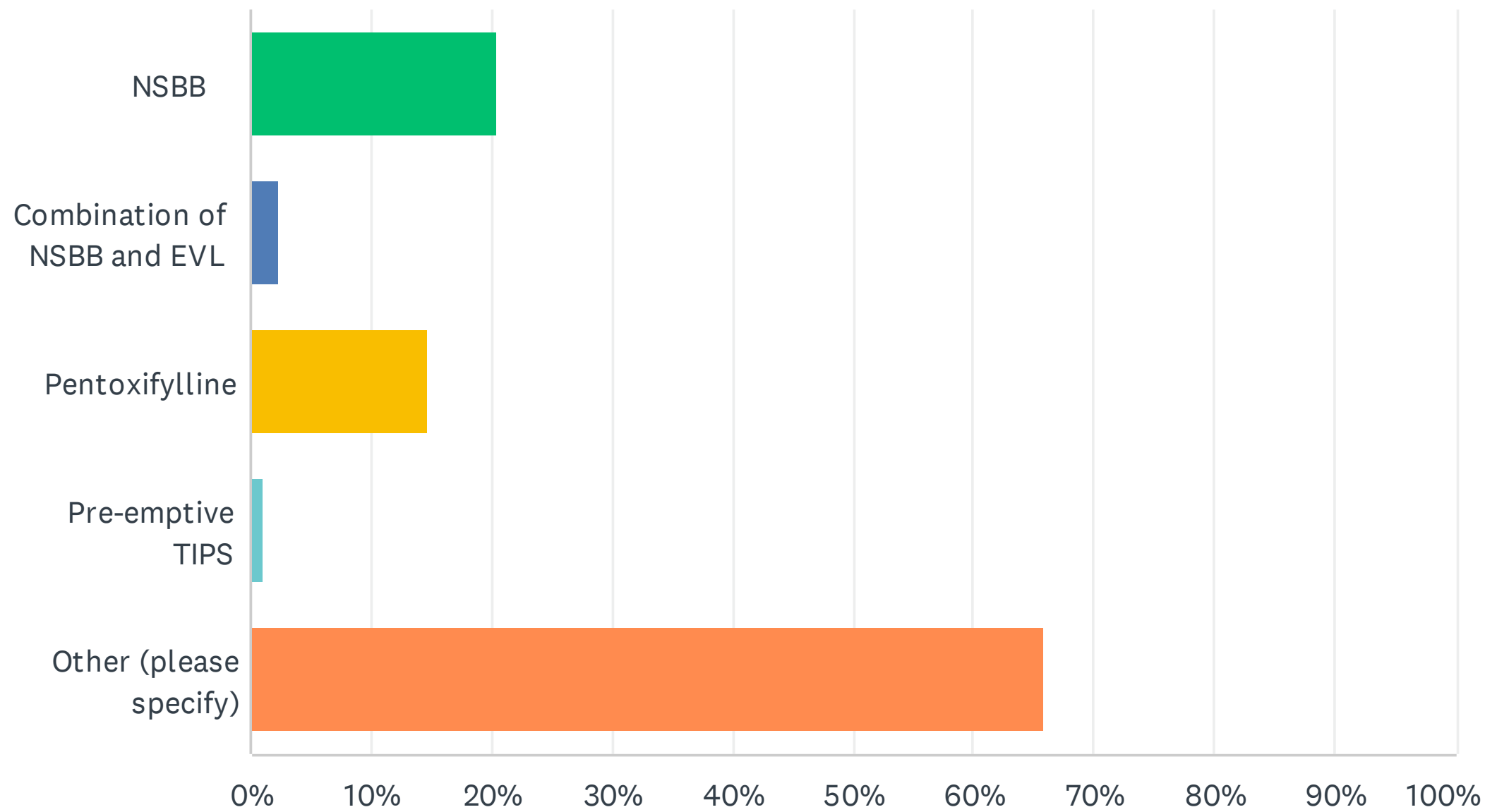

ANSWER CHOICES	RESPONSES	
NSBB	20.45%	18
Combination of NSBB and EVL	2.27%	2
Pentoxifylline	14.77%	13
Pre-emptive TIPS	1.14%	1
Other (please specify)	65.91%	58
Total Respondents: 88		

Q28 In patients with severe alcohol associated hepatitis (MELD score > 20), with no varices, but likely portal hypertension, do you use (Select all that apply)

Answered: 89 Skipped: 25

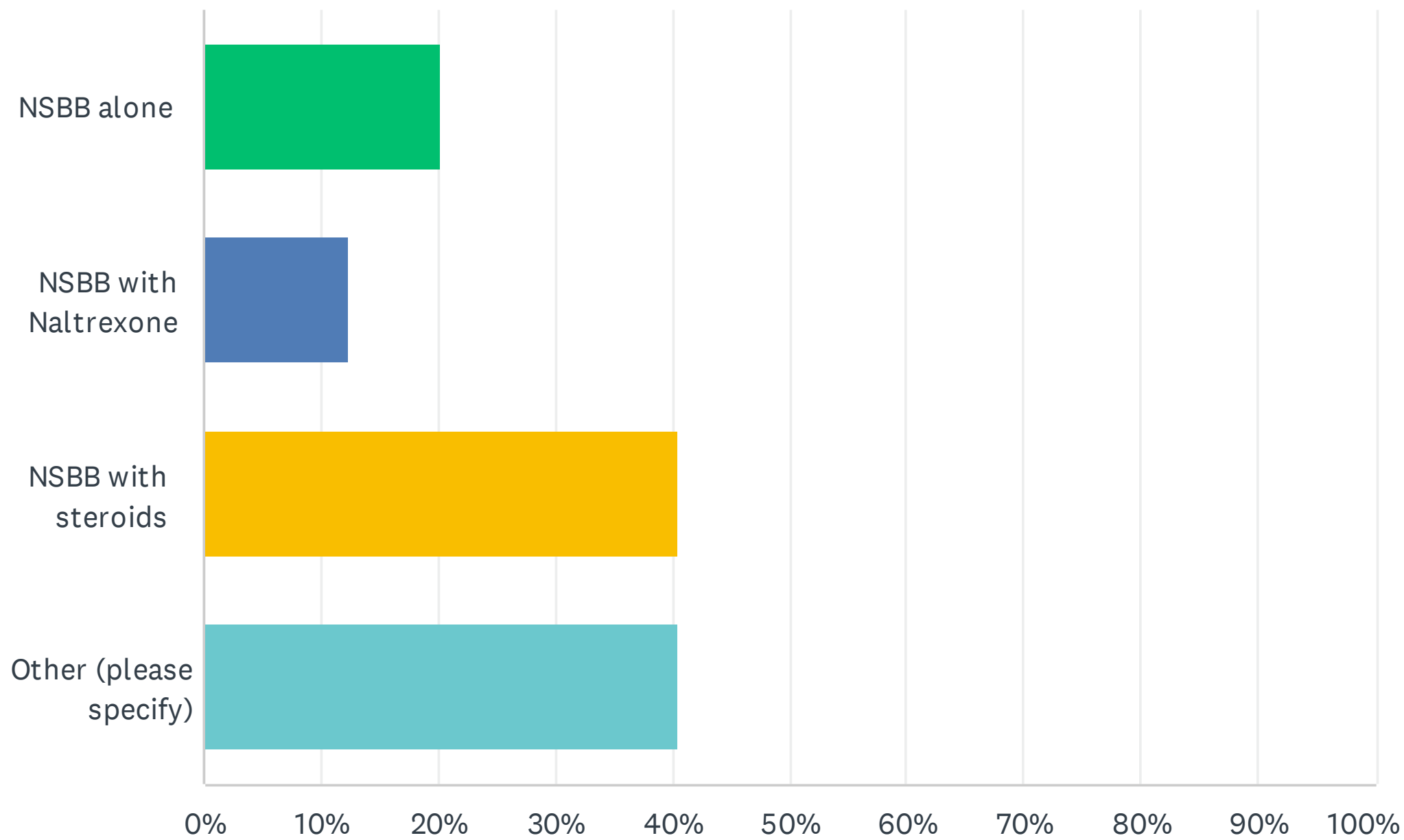

ANSWER CHOICES	RESPONSES	
NSBB alone	20.22%	18
NSBB with Naltrexone	12.36%	11
NSBB with steroids	40.45%	36
Other (please specify)	40.45%	36
Total Respondents: 89		
